# Modelling immune cytotoxicity for cholangiocarcinoma with tumour-derived organoids and effector T cells

**DOI:** 10.1038/s41416-022-01839-x

**Published:** 2022-05-21

**Authors:** Guoying Zhou, Ruby Lieshout, Gilles S. van Tienderen, Valeska de Ruiter, Martin E. van Royen, Patrick P. C. Boor, Luc Magré, Jyaysi Desai, Kübra Köten, Yik Yang Kan, Zhouhong Ge, Lucia Campos Carrascosa, Cecile Geuijen, Dave Sprengers, Luc J. W. van der Laan, Monique M. A. Verstegen, Jaap Kwekkeboom

**Affiliations:** 1 Department of Gastroenterology and Hepatology, Erasmus MC University Medical Center Rotterdam, Rotterdam, The Netherlands; 2 Department of Surgery, Erasmus MC Transplant Institute, Erasmus MC University Medical Center Rotterdam, Rotterdam, The Netherlands; 3 Department of Pathology, Erasmus MC University Medical Center Rotterdam, Rotterdam, The Netherlands; 4 Merus N.V., Utrecht, The Netherlands

**Keywords:** Bile duct cancer, Cancer models, Immunotherapy, Cancer microenvironment

## Abstract

**Background:**

Immunotherapy with immune checkpoint inhibitors (ICIs) is being explored to improve cholangiocarcinoma (CCA) therapy. However, it remains difficult to predict which ICI will be effective for individual patients. Therefore, the aim of this study is to develop a co-culture method with patient-derived CCA organoids and immune cells, which could represent anti-cancer immunity in vitro.

**Methods:**

CCA organoids were co-cultured with peripheral blood mononuclear cells or T cells. Flow cytometry, time-lapse confocal imaging for apoptosis, and quantification of cytokeratin 19 fragment (CYFRA) release were applied to analyse organoid and immune cell behaviour. CCA organoids were also cultured in immune cell-conditioned media to analyse the effect of soluble factors.

**Results:**

The co-culture system demonstrated an effective anti-tumour organoid immune response by a decrease in live organoid cells and an increase in apoptosis and CYFRA release. Interpatient heterogeneity was observed. The cytotoxic effects could be mediated by direct cell–cell contact and by release of soluble factors, although soluble factors only decreased viability in one organoid line.

**Conclusions:**

In this proof-of-concept study, a novel CCA organoid and immune cell co-culture method was established. This can be the first step towards personalised immunotherapy for CCA by predicting which ICIs are most effective for individual patients.

## Background

Cholangiocarcinoma (CCA) is the second most prevalent type of primary liver cancer. It is an aggressive malignancy that originates from the biliary tract [1–3]. As CCA is often asymptomatic until late stages, at the time of diagnosis, 70–80% of the patients have non-curable disease and receive a palliative treatment regimen consisting of chemotherapeutics. Marked heterogeneity makes CCA difficult to treat successfully and the prognosis for CCA patients is poor, with 5-year survival rates of 7–20% [4, 5]. Immunotherapy is one of the avenues that is being explored to improve CCA treatment. Immune checkpoint inhibitors (ICIs) have been shown to be highly effective in activating pre-existing anti-cancer T cell responses in patient subsets with several types of advanced malignancies [6, 7]. Clinical data on the efficacy of these inhibitors in CCA is limited. In phase 2 studies, anti-programmed cell death protein 1 (anti-PD-1) antibody nivolumab demonstrated complete or partial remission (objective response) in 22% of biliary tract cancer patients [8], and nivolumab combined with anti-cytotoxic T lymphocyte antigen 4 antibody ipilimumab induced an objective response in 31% of patients with intrahepatic CCA [9]. Nevertheless, the majority of CCA patients do not respond well to these antibody treatments and it has proven difficult to predict which patients will benefit. Therefore, it would be of great benefit to develop an in vitro model that predicts a patient’s response to specific ICI in order to provide optimal immunotherapy.

The establishment of cancer organoids has had a significant impact on the cancer research field by providing a patient-specific three-dimensional (3D) model designed to better represent the tumour than conventional two-dimensional cell cultures [10–16]. However, cancer organoids only represent the epithelial compartment of the tumour, limiting their utility in several fields of cancer research, including immunotherapy. Therefore, combining tumour organoids with immune cells has become an important objective to create an in vitro model suitable to study their interactions and for the evaluation of immunotherapies. Co-cultures of organoids and immune cells have already been reported for several types of cancer, including gastric cancer organoids co-cultured with autologous CD8^+^ T cells and dendritic cells [17], pancreatic cancer with allogeneic T cells [18], autologous peripheral blood mononuclear cells (PBMCs) [19], and autologous CD8^+^ T cells and dendritic cells [20], rectal cancer with autologous tumour-infiltrating lymphocytes (TILs) [21], and non-small cell lung cancer and colorectal cancer co-cultured with T cells [22].

Patient-derived CCA organoids were established from resected tissue specimens [11, 23–25] and from needle biopsies [26, 27]. Both were shown to largely recapitulate gene expression and mutational patterns of the patient tumour [11, 23, 25–27] and were suitable for high-throughput compound screens that identified several potential treatment options for CCA patients [11, 23, 27]. However, even though CCA organoids have been in use for several years, no co-culture method combining CCA organoids with immune cells has been reported so far.

Therefore, the aim of the current study is to establish and optimise an in vitro 3D co-culture model of patient-derived CCA tumour organoids with PBMCs and purified T cells and to study growth inhibition and induction of organoid cell death by these cells. We performed several readouts for cytotoxicity, including flow cytometry, injury marker release in supernatant, and time-lapse confocal imaging. Furthermore, we investigated the mechanism of cell death induced by T cells and described patient-specific differences in sensitivity to immune cell cytotoxicity. Applications of this novel culture system for screening of new immunotherapeutic antibodies and precision medicine will be discussed.

## Materials and methods

### Human subjects

Fresh tumour tissue samples were collected from seven liver cancer patients after surgical tumour resection at the Erasmus Medical Center Rotterdam. None of the patients received chemotherapy or immunosuppressive therapy at least 3 months prior to the surgery. The use of tumour tissue samples for research purposes was approved by the Erasmus MC medical ethics committee (MEC-2012–331; MEC-2013-143; MEC-2016-375). All patients consented to donate resected materials for research purposes. Peripheral blood buffy coat was obtained from four healthy donors (provided by Sanquin, Amsterdam, the Netherlands). PBMCs were isolated by Ficoll density gradient centrifugation (600 × *g*, 20 min, 20 °C) as described previously [28] and frozen at −150 °C for later use.

### Organoid culture

Organoids were initiated and cultured from three CCA patients (Supplementary Table S1) as described previously [11]. A brief description can be found in Supplementary Materials. Tumorigenicity of CCA organoids was confirmed by tumour formation after subcutaneous injection in mice. Animal experiments were done in accordance to the Dutch law on laboratory animal use and performed under license number AVD101002017867—protocol 17-867-19. Organoid fragments were injected into the flanks of female NOD.Cg-Prkdc^SCID^ Il2rg^tm1Wjl^/SzJ (NSG) mice (Charles River). Mice were checked weekly for tumour growth and tumours were harvested when tumour volume was >2 cm^3^ or organoid fragments were injected >6 months ago. CCA1 tumour formation was found in 3/3 injection sites, CCA2 tumour formation in 4/4 injection sites, and CCA3 in 4/5 injection sites. Tumours were harvested and histology was assessed to confirm CCA-like tissue morphology. Moreover, mutation analysis was performed by targeted next-generation sequencing for 65 cancer-related genes.

### Optimisation of basement membrane extract (BME) concentration in co-culture assay

PBMC or purified CD3^+^ T cells were thawed 1–2 days in advance and pre-activated with anti-human CD3/CD28 dynabeads at a 1:100 bead:cell ratio in T cell medium (TM: RPMI 1640 (Lonza, Breda, The Netherlands) supplemented with 2 mM L-glutamine/Ultraglutamine (Invitrogen, Waltham, MA, USA), 50 mM Hepes Buffer (Lonza), 1% penicillin–streptomycin (Life Technologies), 5 mM sodium pyruvate (Gibco), 1% minimum essential medium non-essential amino acids (Gibco, Waltham, MA, USA), and 10% human AB serum (Invitrogen)). One well of organoids was dissociated into single cells and counted to determine the effector:target cell ratio. Organoids used for co-culture were harvested, washed, and mechanically fragmented. For co-culture without BME, organoid fragments were plated in a flat-bottom 96-well plate. PBMC were added at a 5:1 effector:target cell ratio. For co-culture with BME domes, organoid fragments were plated in a 75% BME dome in a flat-bottom 96-well plate, T cells were resuspended in the medium and were added 1–2 h later around the BME dome at a 20:1 effector:target cell ratio. For co-culture in 10% BME suspension, organoid fragments and PBMCs were resuspended in medium with 10% BME and plated in a flat bottom 96-well plate.

### Bright field and fluorescence imaging

Bright field and fluorescence imaging was performed using an EVOS FL Cell Imaging System outfitted with red fluorescent protein and 4′,6-diamidino-2-phenylindole (DAPI) light cubes. To analyse cell death, organoids and co-cultures were stained before imaging by adding 12.5 µg/ml propidium iodide (Sigma-Aldrich, Saint Louis, MO, USA) and 100 µg/ml Hoechst 33342 to the culture medium for 1 h.

### ATP quantification cell viability assay

ATP quantification was performed to determine the effect of medium composition adjustments on the organoids. Therefore, organoids were mechanically disrupted and split into 5 µl droplets plated in a white-wall 96-well plate. Organoids were maintained in organoid expansion medium, supplemented with 0, 2.5, 5, 7.5, or 10% human serum, supplemented with 0, 20 IU/ml (4 ng/ml) or 100 IU/ml (20 ng/ml) IL-2 or deprived of nicotinamide and/or forskolin for 6–7 days. CellTiter-Glo® 3D Cell Viability Assay (Promega, Madison, WI, USA) was used to quantify ATP content in accordance with the manufacturer’s instructions.

### PBMC proliferation and activity measurement

PBMCs from healthy donors were cultured in TM, organoid medium (OM) with 10% human serum, or OM with 10% human serum without one of the following reagents: A8301, recombinant human [Leu15]-Gastrin I, recombinant human epidermal growth factor, recombinant human hepatocyte growth factor, forskolin, recombinant human FGF10, N2 supplement, *N*-acetyl-l-cysteine, B27 supplement, nicotinamide, Rspo-1 conditioned medium. Anti-human CD3/CD28 dynabeads (Gibco) were added in all conditions except ‘TM’ and ‘OM’ at a bead:cell ratio of 1:100. Cells were cultured in 96-well round-bottom culture plates at 37 °C.

After 4, 7, and 10 days, respectively, PBMCs were harvested and stained with fixable viability dye eFluor 506 (1:500 in phosphate-buffered saline (PBS), eBioscience, Waltham, MA, USA). Cell surface staining was then performed in the dark at 4 °C for 20 min with anti-CD45, anti-CD8, anti-CD4, anti-CD3, anti-HLA-DR, and anti-CD137 antibodies (Supplementary Table S3). Cells were washed and resuspended in fluorescence-activated cell sorting FACS buffer (PBS with 0.1% EDTA, 0.5% NaN_3_, and 1% foetal calf serum). For Ki-67 staining, cells were fixed and permeabilised using the Foxp3 staining buffer set (eBioscience). PBMCs were measured using a FACSCanto II flow cytometer (BD Biosciences, Franklin Lakes, NJ, USA) and analysed using the FlowJo software (version 10.0, LLC).

### HepG2 killing assay

The HepG2 killing assay was performed as described previously [29]. PBMCs were activated with anti-human CD3/CD28 dynabeads at a 1:100 bead:cell ratio for 3 days. PBMC and HepG2-RFPs were combined at a 30:1 PBMC:HepG2-RFP ratio. They were resuspended in either TM or OM without nicotinamide and co-cultured for 7 days.

### Co-culture assay

#### Organoid preparation

Seven days before co-culture, organoids were harvested, mechanically broken, and dissociated into small, 1–4 cell-sized fragments by Trypsin-EDTA (Gibco) dissociation alternated with mechanical disruption (3 cycles of 3 min Trypsin-EDTA at 37 °C). Later, they were plated in 25 µl droplets of 75% BME and maintained in organoid expansion medium, which was refreshed on days 3–4.

#### Immune cell preparation

The CCA organoid cultures used were typed for HLA-A, -B, -C, -DRB1, -DRB345, and DQB1. PBMCs were isolated from donors that were selected based on HLA mismatching with the CCA organoids. In case of PBMC co-culture, PBMCs were thawed 2–3 days in advance at 37 °C, then resuspended and cultured in TM with anti-human CD3/CD28 dynabeads (Gibco-Life Technologies) at a 1:100 bead:cell ratio. In case of T cell co-culture, HLA-mismatched PBMCs were thawed 2–3 days in advance at 37 °C, then T cells were isolated by CD3-positive selection by microbeads according to the manufacturer’s instructions (Miltenyi, Bergisch Gladbach, Germany). Live T cell purity was ≥95%. T cells were cultured for 2–3 days in TM with anti-human CD3/CD28 dynabeads at a 1:20 bead:cell ratio and 20 IU/ml (4 ng/ml) interleukin (IL)-2.

#### Co-culture

On the day of co-culture, one well of organoids was harvested, mechanically broken, and dissociated into single cells by Trypsin-EDTA incubation (15 min at 37 °C). Quantification of single cells from this well was used to determine effector:target cell ratio between immune cells and organoid cells. Immune cells were harvested, counted, and combined with organoids at 20–30:1 (PBMCs) or 25–50:1 (T cells) ratios. Together, they were resuspended in 200 µl organoid expansion medium without nicotinamide (OM-nic) with 10% BME (suspension coating) and 10% human serum and plated in a 96-well flat-bottom suspension plate. In some conditions, 25 IU/ml (5 ng/ml) IL-2, 50 IU/ml (10 ng/ml) IL-2, or 100 IU/ml (20 ng/ml) IL-2 was added. On day 3–4, 100 µl of pre-warmed OM-nic with 10% human serum was carefully added. Readouts were performed on day 7 of co-culture.

### Live cell quantification by flow cytometry

Organoids and immune cells were harvested, washed, and dissociated into single cells by incubation with TrypLE Express (Thermofisher, 3–5 cycles of 5 min at 37 °C), combined with regular resuspension and vortexing. An aliquot was checked microscopically after cycle 3, 4, and 5 to determine when the organoids were fully dissociated. Cells were washed first in PBS and then in Annexin V binding buffer (eBioscience Annexin V apoptosis detection kit), and stained with anti-EpCAM, anti-CD45, anti-CD3, anti-CD8, anti-CD56, anti-CD14, and Annexin V (Supplementary Table S3) for 10–15 min at room temperature in the dark. Cells were washed and resuspended in Annexin V binding buffer and stained with DAPI to mark dead cells. Counting beads (Invitrogen, 123count eBeads) were added to each well prior to flow cytometric recording (MACSQuant, Miltenyi). A strict gating strategy was made to distinguish live CD45^+^ immune cells and live EpCAM^+^ organoid cells (Supplementary Fig. 3A). The analysis was done using the FlowJo software (version 10.0, LLC).

### Cytokeratin subunit 19 fragment (CYFRA) quantification

Culture supernatants were collected on day 7 of the co-culture assay and stored frozen at −20 °C. CYFRA concentration was measured by the Lumipulse® G1200 (Fujirebio, Tokyo, Japan) automated immunoassay analyser using the Lumipulse® G CYFRA immunoreaction cartridges (Fujirebio) according to the manufacturer’s instructions.

### Confocal time-lapse imaging and analysis

T cells were isolated and pre-activated as described above for co-culture assays. On the day of co-culture, they were stained with 1 µM of CellTrace Far Red (Invitrogen) in PBS for 15 min at 37 °C. Organoids were pre-stained with 0.1 µg/ml Hoechst 33342 (Thermofisher) for 3.5 h. Co-cultures were prepared as described above, with addition of 1:1000 caspase 3/7 detection reagent (Invitrogen) and 0.1 µg/ml Hoechst 33342 to the medium. Co-cultures were imaged in time using a Perkin Elmer confocal Opera Phenix High Content screening system equipped with a ×10 air objective (NA 0.3). Imaging settings and analysis details can be found in Supplementary Materials.

### Conditioned medium assay

TIL isolation and preparation of conditioned media from PBMC and TIL is described in Supplementary Materials. Organoids were harvested, mechanically broken, and dissociated into small, 1–4 cell-sized fragments by Trypsin-EDTA dissociation alternated with mechanical disruption (3 cycles of 3 min Trypsin-EDTA at 37 °C). In all, 5 µl droplets of 75% BME (diluted in organoid base medium) with 1500 organoid fragments were plated in a 96-well flat-bottom plate. Each droplet was covered with 100 µl of OM-nic supplemented with 0, 2.5, 5, 7.5, or 10% concentrated control medium, concentrated conditioned medium from stimulated PBMCs or TILs, or concentrated conditioned medium from unstimulated TILs. Medium was refreshed on day 4. ATP quantification by CellTiter-Glo® 3D Cell Viability Assay (Promega) was performed on day 7.

### Analysis of co-stimulatory and co-inhibitory molecules by flow cytometry

Organoids were harvested and dissociated into single cells with TrypLE Express (3 cycles of 5 min at 37 °C), combined with regular resuspension and vortexing. A half of organoid cells were stimulated with 100 ng/ml interferon (IFN)-γ for 2 days, while the other half without stimulation. The culture was in OM in 96-well round-bottom plates at 37 °C. After 2 days, cells were harvested and stained with fluorochrome-conjugated antibodies against different immune molecules in the presence of Fc receptor block (Supplementary Table S3). DAPI was used to mark dead cells, and minus controls were used for gating purposes.

### RNA sequencing analysis

Sample preparation, RNA sequencing details, and data preparation can be found in Supplementary Materials. Data are stored in the GEO repository under accession number GSE179601. Differentially expressed genes were determined by R package Limma (version 3.46.0) comparing CCA1 against CCA2 and CCA3. Gene set enrichment analysis was performed for the 1000 genes with the highest ^2^log(fold change) using the publicly available online analysis tool DAVID [30, 31] with the Gene Ontology: Biological Processes database. Processes with false discovery rate (FDR)-adjusted *p* value <0.25 were deemed significantly enriched.

### Statistical analysis

Differences among multiple matched groups of data were analysed by either one-way analysis of variance or mixed-effects analysis depending on missing values. Differences between two matched groups of data were analysed by two-tailed paired *t* test. Differences between two unmatched groups of data were analysed by two-tailed unpaired *t* test. The statistical analyses were performed using GraphPad Prism 9 (GraphPad Software). *p* Values <0.05 were considered statistically significant (*p < 0.05; **p < 0.01; ***p < 0.001).

## Results

### Establishment and optimisation of 3D co-culture conditions

**Fig. 1:**
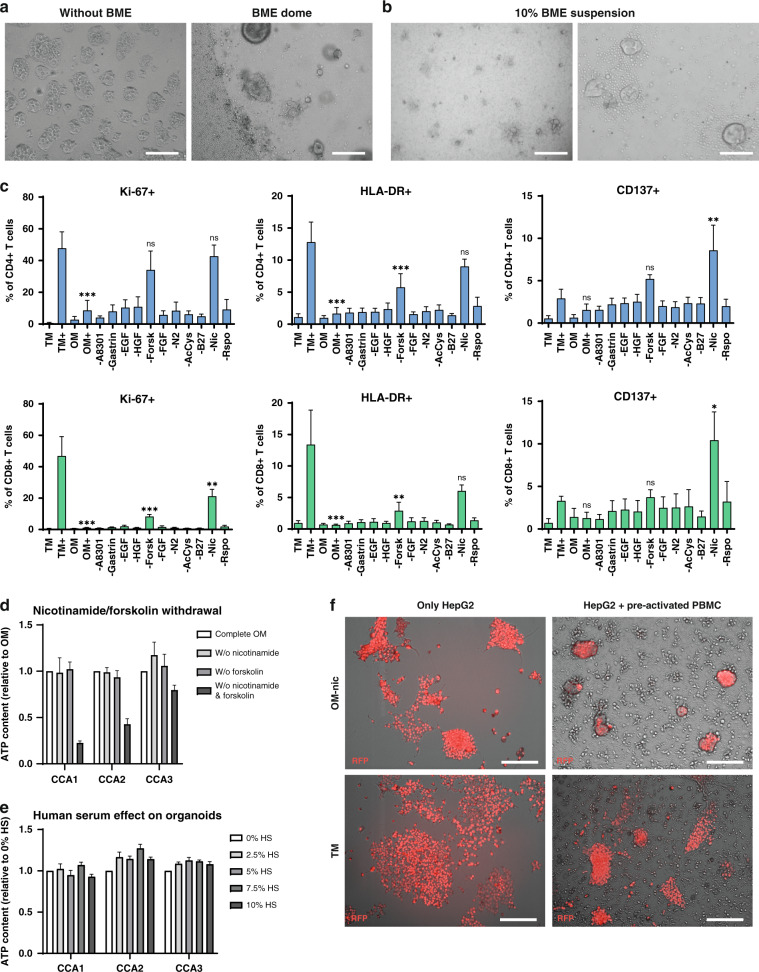
Optimisation of the co-culture setup for CCA organoids and immune cells. Bright field images on days 4–5 show that CCA organoids lose 3D morphology in culture without BME and PBMCs are not able to interact well with CCA organoids in BME domes (**a**). Co-culture in a 10% BME in medium suspension (**b**) can sustain 3D organoid morphology and allows PBMC–organoid interaction. CCA organoids and PBMCs were exposed to different medium compositions to find a medium in which both survive and function. Flow cytometry for Ki-67, HLA-DR, and CD137 protein expression in CD4^+^ and CD8^+^ T cells after 7 days of culture in T cell medium without and with anti-CD3/CD28 coated beads (TM, TM+), organoid medium without and with anti-CD3/CD28 coated beads (OM, OM+), and organoid medium without specified components in the presence of anti-CD3/CD28 coated beads (**c**) demonstrates an inhibitory effect of forskolin and nicotinamide on T cells (*n* = 3 different PBMC donors). Statistical significance is depicted for OM+, -forsk and -nic compared to TM+. ATP quantification shows that CCA organoid survivability is not affected by removal of forskolin or nicotinamide from the organoid medium, but removal of both decreases viability (**d**) (*n* = 3 technical replicates). CCA viability was not affected by the addition of human serum to organoid medium as determined by ATP quantification assay (**e**) (*n* = 3 technical replicates). Overlay of bright field (grey scale) and RFP (red) demonstrates that the killing potency of anti-CD3/CD28 bead pre-activated PBMCs in both T cell medium (TM) and organoid medium without nicotinamide (OM-nic) is comparable (**f**). Scale bar: 500 µm (**a** and left panel of **b**), 100 µm (right panel of **b**), 200 µm (**f**). All values with error bars represent mean with SEM. *p < 0.05, **p < 0.01, ***p < 0.001.

To set up a 3D co-culture model, we used established patient CCA organoid lines and PBMCs from healthy donors to establish co-culture conditions in which both could survive and function. First, we compared co-culture of organoid fragments and PBMC without BME to co-culture in which organoid fragments were cultured in BME domes while PBMCs were in the medium around the dome (Fig. 1a). Without BME, organoids adhered to the plastic did not propagate well and could not retain their 3D morphology. Loss of the 3D self-organisation of organoids limits cellular interactions and changes cellular behaviour. Therefore, co-culture without BME was not feasible. Culture in BME domes limited interaction between organoids and PBMC, as only a small part of the PBMC could infiltrate into the BME dome over a short distance in this setting. To overcome this, we suspended the organoid fragments and PBMCs in medium with 10% BME (Fig. 1b). This way, CCA organoids kept their 3D morphology and PBMCs gathered around the organoids.

Consecutively, we examined the influence of TM on organoid morphology and that of OM on T cell proliferation and activation. CCA organoids were not able to retain their 3D morphology in TM (Supplementary Fig. S1A), while CD4^+^ and CD8^+^ T cells displayed reduced proliferation (Ki-67^+^ cells) and activation (HLA-DR^+^ cells) when cultured in OM (CD4^+^ Ki-67^+^ 8.6% in OM+ vs 47.7% in TM+, *p* < 0.001 and HLA-DR^+^ 1.7 vs 12.8%, *p* < 0.001; CD8^+^ Ki-67^+^ 1.1 vs 46.8%, *p* < 0.001 and HLA-DR^+^ 0.6 vs 13.4%, *p* < 0.001) (Fig. 1c). So, we eliminated components of the OM to identify their contribution to the observed suppression of T cell activity. Of all the components investigated, removal of forskolin partially and removal of nicotinamide almost completely abolished the suppressive effect of OM on T cells and removal of nicotinamide even increased the number of CD137^+^ cells (CD4^+^ CD137^+^ 10.4% in OM-nic vs 3.3% in TM+, *p* < 0.01; CD8^+^ CD137^+^ 8.6 vs 2.9%, *p* < 0.05) (Fig. 1c and Supplementary Fig. S2). The morphology and viability of all three CCA organoid lines were not affected by removing either nicotinamide or forskolin from the medium (Fig. 1e and Supplementary Fig. S1B), but removal of both reduced organoid viability as expressed by ATP quantification (fold decrease CCA1: 0.23, CCA2: 0.43, CCA3: 0.80; Fig. 1d). The other factor new to T cell culture is BME. In all, 10% BME did not increase the expression of activation markers HLA-DR, CD137, and proliferation marker Ki-67 in both CD4^+^ and CD8^+^ T cells, thereby not leading to unwanted T cell reactivity (Supplementary Fig. S1C). As human serum is a key component in TM, we investigated whether addition to organoid culture would have harmful effects. Human serum did not suppress growth or viability of CCA organoids as shown by stable ATP content at concentrations up to 10% (Fig. 1e).

We tested the cytotoxic properties of PBMCs in OM without the addition of nicotinamide in our previously established model of immune cell-mediated killing of tumour cell line HepG2 [29]. PBMCs were pre-activated with anti-CD3/CD28-coated beads, then co-cultured for 7 days with HepG2 cells that were genetically labelled with RFP. Figure 1f shows that HepG2 cell number was decreased in PBMC co-culture compared to HepG2 alone in both OM without nicotinamide and TM, indicating similar killing efficiency. All together, we determined that culture conditions for the 3D co-culture of CCA organoids and PBMC should consist of OM without nicotinamide, supplemented with 10% human serum, in a 10% BME suspension.

### CCA organoids show interpatient variation in PBMC-mediated cytotoxicity

**Fig. 2:**
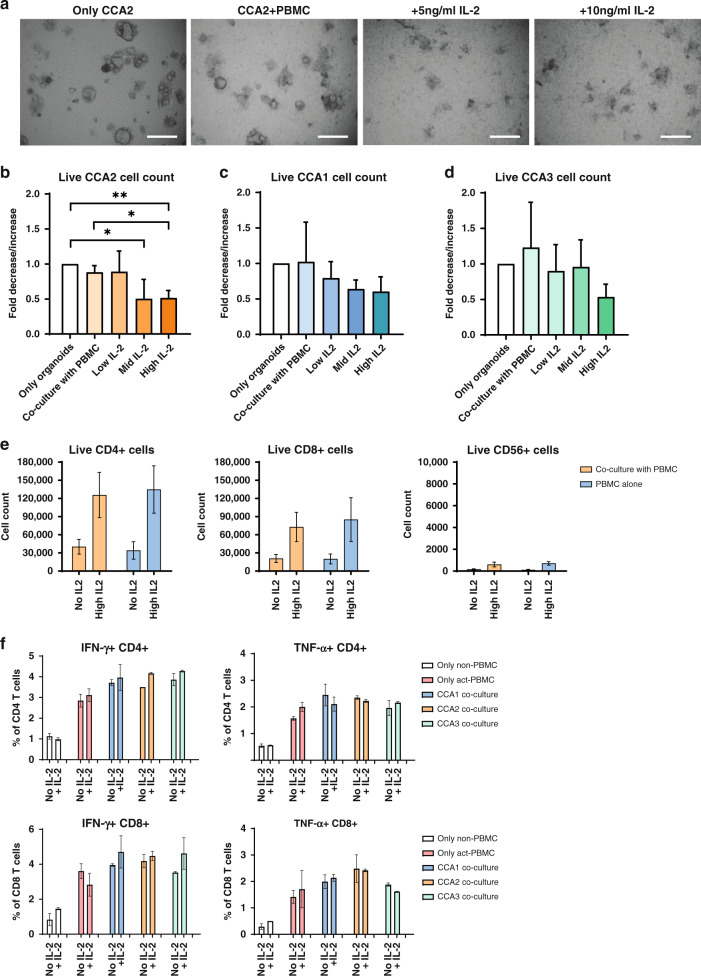
Pre-activated PBMCs co-cultured with CCA organoids and IL-2 show patient-specific killing. CCA2 organoids were co-cultured with anti-CD3/CD28 pre-activated PBMCs for 7 days. Bright field imaging (**a**) shows that CCA2 organoids lose their regular morphology and become fragmented, especially upon addition of IL-2. Flow cytometry cell count for EpCAM-positive and CD45- and DAPI-negative cells (**b**) demonstrates that CCA2 organoids sustain significant cell death in the presence of 50 IU/ml (10 ng/ml, mid) or 100 IU/ml (20 ng/ml, high) IL-2, but not with 25 IU/ml (5 ng/ml, low) IL-2. CCA1 (**c**) and CCA3 (**d**) organoids do not show a significant decrease in live cells in the same co-culture setting (*n* ≥ 3 biological replicates for each organoid line; *n* = 2 different PBMC batches). Flow cytometry cell count for live (DAPI-negative) CD4^+^ and CD8^+^ T cells and CD56^+^ NK cells demonstrates that the number of live cells is comparable between solo culture and co-culture and addition of 100 IU/ml IL-2 increases the number of live cells (**e**). Flow cytometry for intra-cellular staining of IFN-γ and TNF-α in non-pre-activated and 3-day pre-activated PBMC cultures, and co-cultures of CCA organoids with pre-activated PBMCs, either with or without 100 IU/ml IL-2 (**f**). The percentage of cytokine-positive CD4^+^ and CD8^+^ T cells was determined after 19 h of (co-)culture in the presence of brefeldin and monensin (*n* = 3 technical replicates). Scale bar: 500 µm (**a**). All values with error bars represent mean with SEM. *p < 0.05, **p < 0.01.

Next, CCA organoids were co-cultured with HLA-mismatched anti-CD3/CD28 bead pre-activated PBMCs for 7 days. IL-2 is known to stimulate T cell proliferation and activation, so we investigated whether the addition of three concentrations of IL-2 could enhance the effect of immune cells in co-culture, as IL-2 alone did not affect the viability of CCA organoids (Supplementary Fig. S3B). In co-culture, morphological changes in CCA2 like loss of 3D structural integrity indicated cytotoxicity of PBMCs, which seems to aggravate upon addition of 5 and 10 ng/ml IL-2 (Fig. 2a). Flow cytometry (Fig. 2b) shows that co-culture with PBMCs seemed to lower the number of live organoid cells (EpCAM^+^ DAPI^−^ cells) in CCA2 (fold decrease 0.89, *p* > 0.05), although not significantly. Addition of 10 or 20 ng/ml IL-2 significantly lowered the number of live organoid cells (fold decrease 0.50, *p* < 0.05 and 0.51, *p* < 0.01). The number of live cells in CCA1 and CCA3 was not significantly reduced by co-culture with PBMCs alone (Fig. 2c, d). While IL-2 also potentiated PBMC cytotoxicity in these organoid lines, their decrease in live cells was not significant. IL-2 increased the live cell count of CD4^+^ T cells, CD8^+^ T cells and CD56^+^ natural killer (NK) cells in both the co-culture and the single PBMC culture (Fig. 2e). The number of CD8^+^ T cells was over 100-fold higher and the number of CD4^+^ T cells was about 200-fold higher than the NK cell counts. After pre-activation with anti-CD3/CD28 beads, CD4^+^ and CD8^+^ T cells clearly produced IFN-γ and tumour necrosis factor (TNF)-α compared to non-pre-activated T cells (Fig. 2f). Co-culture with CCA organoids for 19 h seemed to already further enhance the production of these effector cytokines by CD4^+^ and CD8^+^ T cells, although at a low level. Greater effects might be observed if the timeline of the co-culture is prolonged before IFN-γ and TNF-α measurement. These data suggest that this optimised co-culture system allows for studying the interaction between CCA organoids and PBMCs and for quantification of immune cell cytotoxicity of the cancerous organoid cells.

### Purified T cells recapitulate the PBMC-mediated cytotoxicity

**Fig. 3:**
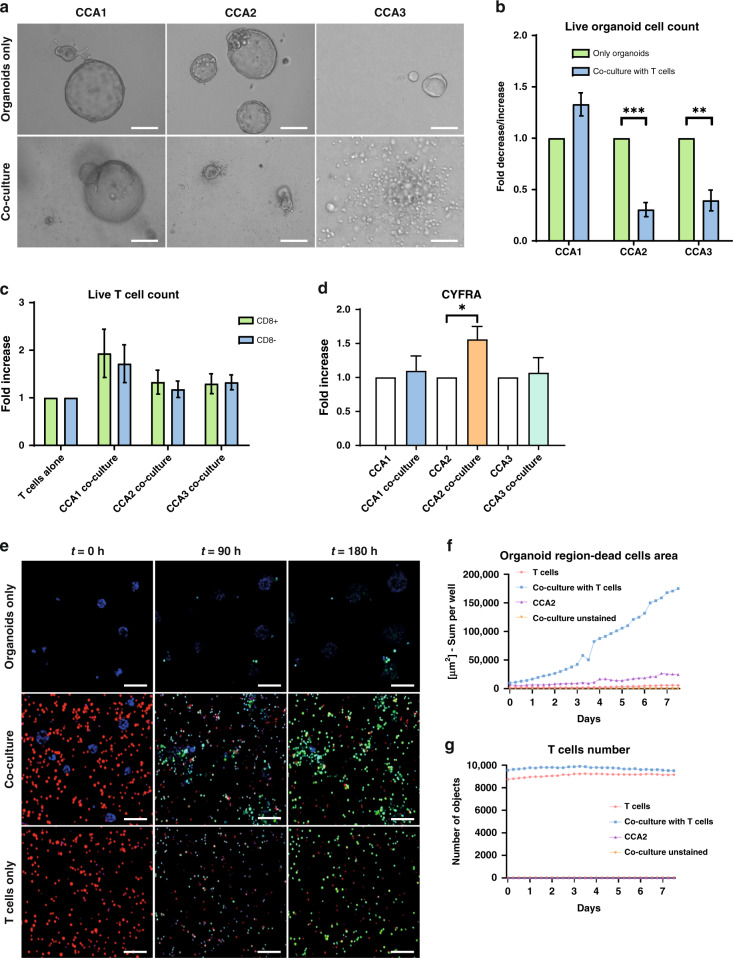
Pre-activated T cells also kill CCA organoids in a patient-specific manner. Bright field images (**a**) of CCA organoids cultured with and without T cells for 7 days demonstrate that CCA1 organoids are not inhibited by T cells while CCA2 and CCA3 organoids are smaller and disintegrated, indicating cell death. Flow cytometric cell count for EpCAM-positive, CD45-negative, and DAPI-negative cells (**b**) shows that CCA2 and CCA3 organoids sustain significant cell death after 7 days of co-culture with anti-CD3/CD28 bead pre-activated T cells, while CCA1 organoids are not affected (*n* ≥ 3 biological replicates for each organoid line; *n* = 2 different PBMC batches). Flow cytometric cell count for live (DAPI-negative) CD8^+^ and CD8^-^ T cells (**c**). Relative difference in CYFRA concentration in supernatant after 7 days of CCA organoid and T cell co-culture (**d**) demonstrates a significant increase in CCA2 (*n* ≥ 3 biological replicates for each organoid line). Representative confocal images of Cell Trace Far Red-stained T cells (red), Hoechst 33342-stained CCA2 organoids (blue), and co-cultures in the presence of a Caspase 3/7-probe (green) at timepoints 0, 90, and 180 h (**e**). Quantification of dead cell surface (Caspase 3/7 probe-positivity) in organoid area (Hoechst 33342 labelled) (**f**) and number of T cells (**g**) in nine fields of view of confocal time-lapse imaging every 6 h for 180 h. Organoid death is higher in co-cultures with T cells compared to CCA2 organoids alone. The number of T cells (Cell Trace Far Red labelled) is comparable between solo culture and co-culture. Scale bar: 200 µm (left and middle panel of **a**), 50 µm (right panel of **a**), 100 µm (**e**). All values with error bars represent mean with SEM. *p < 0.05, **p < 0.01, ***p < 0.001.

As PBMC are a mixed population of cells, we subsequently investigated whether purified T cells were able to kill CCA organoids. CD3^+^ T cells (purity ≥95%) were isolated from HLA-mismatched PBMCs, pre-activated with anti-CD3/CD28-coated beads and co-cultured with CCA organoids. After 7 days of co-culture, CCA1 organoids were not affected morphologically, while CCA2 and CCA3 organoids were smaller and partially disintegrated (Fig. 3a), suggesting cell death. Quantification of live cells showed significant CCA organoid cell death in co-culture with T cells in both CCA2 (fold decrease 0.30, *p* < 0.001) and CCA3 (fold decrease 0.39, *p* < 0.01) co-cultures, whereas CCA1 organoid live cell counts were not affected (Fig. 3b). Co-culture with CCA organoids did not reduce the live cell count of cytotoxic (CD8^+^) and CD8^-^ T cells compared to their single culture (Fig. 3c). CYFRA is a cytokeratin 19 fragment that is usually determined in serum or plasma as a (potential) biomarker for a range of different solid tumours, including CCA [32, 33]. It is released from apoptotic and necrotic cholangiocytes, while it is not released by immune cells [34]. Hence, we hypothesised that it might serve as a simple and scalable readout in our co-culture system. CYFRA concentration was higher in the culture supernatant of CCA2 co-culture than in the culture supernatant of only organoids (fold increase 1.56, *p* < 0.05), while it was not significantly increased in CCA1 and CCA3 co-cultures (Fig. 3d and Supplementary Fig. S3C). Confocal time-lapse imaging showed that more CCA2 organoid cells were caspase 3/7 probe positive in the co-culture with T cells compared to the culture of only organoids (Fig. 3e and Supplementary Video S1 vs S2), indicating increased apoptotic cell death in co-cultured organoids. T cell only culture also showed that a large number of T cells is caspase 3/7 probe positive (Fig. 3e and Supplementary Video S3), which could be due to apoptosis, but is also seen upon T cell activation with anti-CD3 [35]. Quantitative analysis of the confocal images confirmed that the apoptotic cell area in the organoid region was increased about sevenfold after 7 days of co-culture with T cells compared to the single culture of organoids (Fig. 3f). The number of T cells was similar in the co-culture and in single culture (Fig. 3g). Together, these data indicate that strong killing of CCA organoids mediated by T cells is possible in our co-culture system and the degree of cytotoxicity is different between CCA organoid lines.

### Soluble immune cell-mediated factors exert pleiotropic effects on CCA organoids

**Fig. 4:**
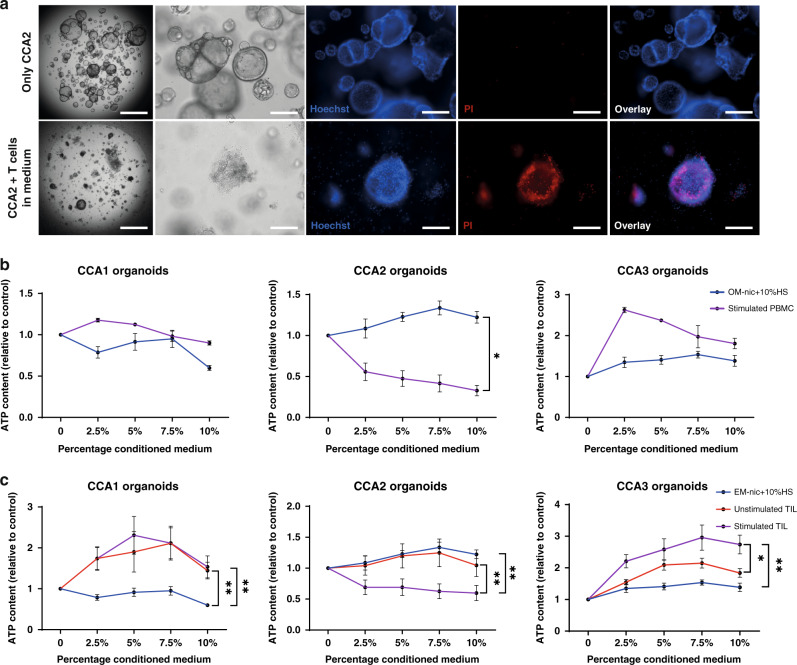
PBMCs and TIL have patient-specific indirect CCA organoid killing capacity. Representative bright field images (first two panels) and immunofluorescent staining with Hoechst 33342 (blue) and propidium iodide (red) of CCA organoids alone (top row) and co-culture of CCA organoids inside the BME dome with T cells surrounding the dome (bottom row) demonstrate CCA organoid death is increased in the co-culture setting (**a**). Viability measurements show the effect of vehicle medium (OM –nic +10% human serum), stimulated PBMC (**b**), unstimulated and stimulated TIL (**c**) conditioned medium on CCA organoids after 7 days (*n* ≥ 3 biological replicates with different PBMC donor cultures or TIL patient cultures to produce conditioned media). The conditioned media and vehicle medium were concentrated 10-fold using 3 KDa centrifugal filters. CCA2 organoid viability is lowered by both conditioned media, while CCA1 and CCA3 are not inhibited by PBMC conditioned medium and stimulated by TIL-conditioned media. Scale bar: 1000 µm (first panel of **a**), 200 µm (other panels of **a**). All values with error bars represent mean with SEM. *p < 0.05, **p < 0.01.

In the co-culture experiments in which organoids were cultured in BME domes, while T cells were added in the medium around the dome, T cells were hardly able to infiltrate. Still, CCA2 organoids were killed after 7 days of co-culture in this setting (Fig. 4a). This suggests that T cells might be able to kill CCA organoids without direct contact, possibly mediated by soluble factors produced by T cells. Therefore, we also studied the indirect effect of soluble factors produced by immune cells on CCA organoids. For this, supernatant was collected from stimulated PBMC (stimulated PBMC-conditioned medium) and control medium (OM-nic with 10% human serum) and concentrated 10 times by centrifugal filtration before adding to the culture. CCA organoids were cultured in BME domes surrounded by medium supplemented with different amounts (2.5, 5, 7.5, and 10%) of concentrated conditioned medium or control medium. The viability of CCA2 organoids was significantly suppressed by stimulated PBMC-conditioned medium compared to control medium (fold decrease 0.33 vs 1.22 at 10%, *p* < 0.05) after 7 days (Fig. 4b). Yet, CCA1 and CCA3 organoids did not show growth inhibition. Next, specific TIL-derived soluble factors were tested by adding concentrated conditioned media from unstimulated and stimulated TIL to CCA organoid cultures. Similarly, stimulated TIL-conditioned medium suppressed the viability of CCA2 organoids compared to both unstimulated TIL-conditioned medium and control medium (fold decrease stim. TIL: 0.60 vs unstim. TIL: 1.05 vs control: 1.22 at 10%, *p* < 0.01) (Fig. 4c). Interestingly, both unstimulated and stimulated TIL-conditioned media increased the viability and/or proliferation of CCA1 (fold increase stim. TIL: 1.54 vs unstim. TIL: 1.44 vs. control: 0.60 at 10%, *p* < 0.01) and CCA3 (fold increase stim TIL: 2.74 vs unstim. TIL: 1.84 vs control: 1.38 at 10%, *p* < 0.05) organoids. These data reveal that the soluble factors secreted by stimulated TIL and PBMC show CCA organoid line-specific effects, ranging from the induction of cell death in CCA2, through TIL-specific stimulation in CCA1 and CCA3.

### Variation in immune checkpoint expression in CCA organoids

The described results demonstrate clear distinctions in the response of different patient-derived organoid lines to direct and indirect contact with immune cells. CCA1 organoids were resistant to inhibitory effects in both direct co-culture and indirect conditioned media experiments. In contrast, CCA2 organoids were sensitive to killing in both assays. CCA3 organoids were sensitive to T cell-mediated cytotoxicity in co-culture but were in fact stimulated by TIL-conditioned media. In order to understand the marked variation in CCA organoid response to direct and indirect immune cell contact, the heterogeneity of expression of co-inhibitory and co-stimulatory immune molecules and RNA expression patterns were assessed.

**Fig. 5:**
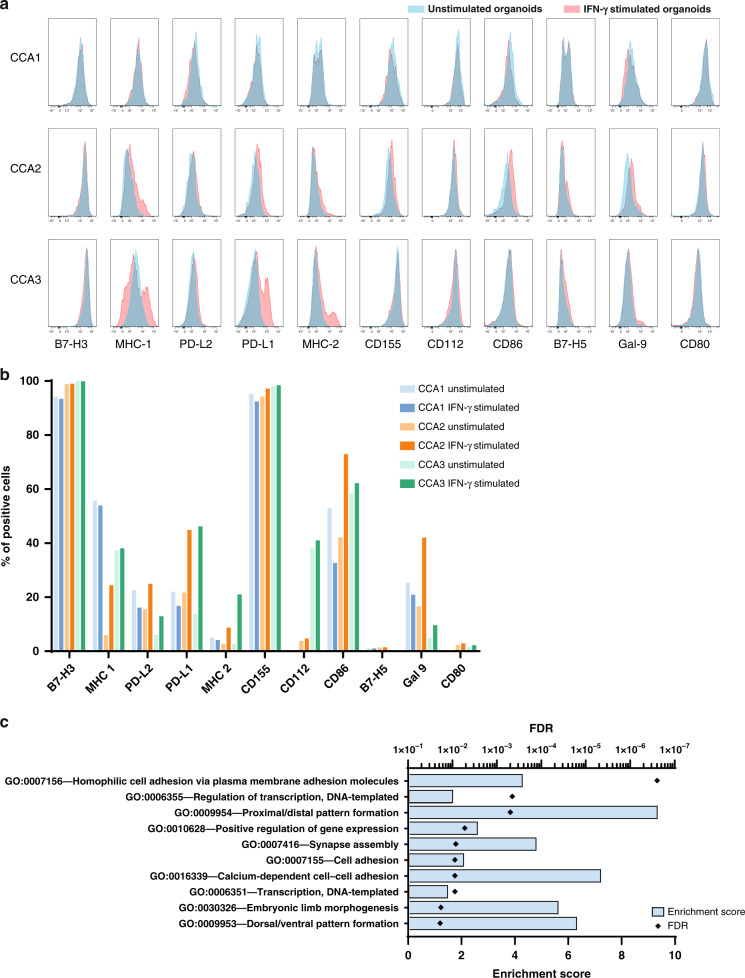
Identifying heterogeneity in immune checkpoint molecule expression and transcriptomics in CCA organoids. Flow cytometry was performed to determine the expression of co-inhibitory and co-stimulatory immune molecules in CCA organoid lines. Histograms show expression on three CCA organoid lines without (blue) or with (red) IFN-γ stimulation (**a**). Results depicted as the percentage of cells expressing the proteins in organoids without and with IFN-γ stimulation (**b**). RNA sequencing data of CCA1 was compared to CCA2 and CCA3 to determine differentially expressed genes. Top 1000 genes with the highest ^2^log (fold change) were inserted into the DAVID tool to perform gene set enrichment analysis using the Gene Ontology: Biological Processes (GO:BP) database. The 10 GO:BP with the lowest FDR are displayed (**c**). Blue bars represent enrichment scores, black diamonds represent FDR values.

The three CCA organoid lines express similar levels of checkpoint molecule B7-H3 (CD276) and CD155 (possible therapeutic immunological target), which was expressed on almost every cell (B7-H3: 94.2–99.9%, CD155: 94.3–98.1%), while major histocompatibility complex (MHC) class 2 molecules (2.6–5.0%), B7-H5 (0.5–1.4%), and co-stimulatory molecule CD80 (0.1–2.2%) were hardly present on CCA organoids (Fig. 5a, b and Supplementary Fig. S4). Co-stimulatory MHC class 1 molecules (5.9%) and CD86 (42.1%) expression was lower in CCA2 organoids than in CCA1 and CCA3. Almost 40% of CCA3 organoid cells are CD112-positive, while CCA1 and CCA2 cells hardly express it (0–3.8%). Co-inhibitory PD-L1 (13.8%) and PD-L2 (6.0%) showed lower expression on CCA3 organoids than in CCA1 and CCA2. Galectin 9 was most present in CCA1 organoids (25.4%), followed by CCA2 (16.6%). After IFN-γ stimulation (Fig. 5a, b and Supplementary Fig. S4), CCA2 and CCA3 organoids upregulated multiple co-stimulatory and co-inhibitory molecules, including PD-L1, PD-L2, MHC class 2 molecules, and galectin 9, and CCA2 organoids also upregulated MHC class 1 molecules and CD86. In turn, CCA1 organoids did not respond to IFN-γ stimulation with upregulation of any of these molecules.

To identify differences in the transcriptome of CCA1 that could potentially explain its resistance to immune-mediated cell death compared to the other CCA organoid lines, we performed gene set enrichment analysis for the 1000 most differentially expressed genes when compared to CCA2 and CCA3. As shown in Fig. 5c, three cell adhesion-related processes were significantly enriched in the gene set, with enrichment scores of 4.2 (GO:0007156, FDR < 0.001), 2.0 (GO:0007155, FDR < 0.01), and 7.1 (GO:0016339, FDR < 0.01). The main type of cell adhesion-related genes found was (proto)cadherins (Supplementary Table S4). Together, these data demonstrate the marked heterogeneity among different patients’ liver tumour organoids in the expression of co-stimulatory and co-inhibitory molecules and RNA expression patterns.

## Discussion/conclusion

CCA is a heterogeneous cancer with a poor prognosis and limited treatment options. Immune therapy is a promising new strategy, and the limited number of clinical trials performed suggest that a subpopulation of CCA patients could benefit from ICI treatment. However, stratification of these patients is challenging. Here we are the first to establish a co-culture model of CCA organoids and immune cells that could potentially serve as an in vitro personalised model to test ICI. This novel 3D co-culture system allows for (1) maintaining the morphology and growth of CCA organoids, (2) survival and function of immune cells, (3) interaction between organoids and immune cells, and (4) quantifiable patient-specific cytotoxic effects of immune cells in CCA organoids. Our work is also the first to demonstrate both cellular contact-based cytotoxicity and soluble factor-mediated cytotoxicity of tumour organoids induced by immune cells.

CCA organoids were cultured with mismatched PBMCs, which resulted in a variable cytotoxic effect. Addition of IL-2 to the cultures enhanced organoid cell death, demonstrating that immune cell function can be modulated in the newly developed co-culture system. The number of CD4^+^ and CD8^+^ T cells far exceeded the number of NK cells in these cultures. We suspected that T cells might be responsible for the majority of the cytotoxic effect, despite different expression levels of MHC class 1 molecules on the three organoid lines. Therefore, CCA organoids were combined with purified CD3^+^ T cells to analyse their specific effect. This confirmed that T cells have potent cytotoxic effects on most but not all CCA organoid lines. The stronger killing effect of T cells compared to PBMCs was probably caused by higher actual effector cell (T cell)/target cell ratios.

In addition to conventionally used flow cytometric and time-lapse confocal analysis to quantify cell death, we aimed to find an easy, quick, and scalable readout by employing a CYFRA quantification assay. The increase in CYFRA determined in the co-culture supernatant of CCA2 with T cells indicates organoid cell death in concordance with flow cytometry and confocal imaging. However, even though similar levels of cell death were found for CCA3 by flow cytometric analysis, there was no clear increase in CYFRA concentration. A possible explanation could be that the number of CCA3 organoid cells in (co-)culture on day 7 was too low, which could be attributed to the lower proliferation rate of these organoids or efficient killing by immune cells at an earlier stage. Consequently, the amount of CYFRA released into the supernatant was too low to measure a significant increase.

Besides the effect that immune cells have on cell viability when cultured in direct cellular contact with tumour organoids, we also demonstrated that soluble factors produced by immune cells have an effect on CCA organoid growth. In detail, one of the organoid lines was susceptible to cell death by soluble factors, while the other two organoid lines displayed induced proliferation by TIL conditioned medium. CD8^+^ cytotoxic T cells are known to produce granzymes and perforins to induce apoptosis in cancer cells [36], which could explain the cytotoxic effects seen in the organoids. In addition to this, T cells produce a range of cytokines supported by CD4^+^ T helper cells, including TNF-α, IFN-γ, and IL-2 [37]. These cytokines could play a part in the inhibition or stimulation of CCA organoids. It would be interesting to further investigate what soluble factors specifically cause cell death in some CCA organoids and what soluble factors cause the acceleration of growth in others.

In general, both direct cellular contact with immune cells and soluble factors produced by immune cells seem to induce a marked heterogeneity in CCA patient organoid responses. CCA tumours are known to be notoriously heterogeneous, displaying a wide range of genomic and epigenetic abnormalities and molecular patterns [4]. Therefore, their biological behaviour is diverse, and variation in immune cell interactions is to be expected. Studies in which rectal cancer organoids were co-cultured with TIL also showed patient-specific differences in immune killing [21]. The factors determining effectivity of immune cell-based killing and immunotherapy in patients are complex and poorly understood. To identify contributing factors to the differences in susceptibility to killing of organoids in our co-culture system, we analysed the protein expression levels of immune co-stimulatory and co-inhibitory molecules and differences in transcriptomes. Although considerable variation in protein expression of immune molecules and response to IFN-γ stimulation was found among the CCA organoid lines, we could not find a direct link to the diversity in response. Gene set enrichment analysis of genes that were expressed in higher levels in CCA1 identified three cell adhesion processes. (Proto)cadherins were the most abundant adhesion genes enriched in these processes. E-cadherin is the best studied cadherin and is known to interact with dendritic cells, macrophages, NK cells, and T cells [38]. However, e-cadherin was not found to be overexpressed here and it remains unclear whether the identified (proto)cadherins are able to modify immune cells, and, if so, whether they would be stimulatory or inhibitory. This does not provide further evidence helping to explain the described differences in susceptibility to immune cell-related cytotoxicity specifically. Thus, further studies are needed using more patient-derived organoid lines to elucidate mechanisms adopted by CCA organoid cells to evade immune cell killing.

The implementation of immune cells in CCA organoid cultures, which consist of epithelial cancer cells only, has provided an important step towards a more complex representation of the tumour microenvironment. The combination of patient-derived CCA organoids and autologous tumour-infiltrating immune cells potentially provides a patient-specific model to determine which ICI could benefit the patient. Acquisition of matched CCA organoids and PBMC or TIL from large patient cohorts remains challenging. CCA organoid research is still at an early stage, and establishment of confirmed CCA cultures is not successful for all patients, with a reported establishment rate of approximately 36% [39]. Moreover, the number of TIL required for the current co-culture protocol demands larger pieces of tumour tissue for isolation, limiting TIL co-cultures to the relatively small subset of CCA patients undergoing surgical resection. Nonetheless, the feasibility of autologous cancer organoid and TIL co-culture has been demonstrated for rectal cancer organoids. In this study, three co-cultures of rectal cancer organoids with TIL were treated with PD-1 antibody pembrolizumab and marked differences in effectivity were observed. No effect was found in one organoid line, temporary improvement was shown for the second, and improved cytotoxicity was only detected in the third organoid line [21]. This study also revealed that organoids established from patients with a complete pathological response to neo-adjuvant chemoradiotherapy showed higher sensitivity to TIL-mediated killing in vitro, showing evidence of clinical relevance of organoid immune cell co-cultures. Subsequently, studies need to be designed to investigate the predictive value of this in vitro model by comparing patient response to ICI to the effect of these ICI in co-cultures of CCA organoids and autologous TIL.

In conclusion, this study provides an optimised an innovative in vitro 3D co-culture method in which patient-derived CCA organoids are cultured with immune cells. Assessing cell death using this method demonstrated quantifiable direct and indirect anti-tumour organoid immune responses that vary between CCA organoid lines. As proof of principle, and likely supplemented with patient-specific immune cells, the co-culture method presented in this study could serve as a useful tool to examine the efficacy of new ICI and to predict which, if any, ICI would be most effective in individual patients.

## Supplementary information

Supplementary Materials

Supplementary Video S1

Supplementary Video S2

Supplementary Video S3

Reproducibility checklist

Approval of author list adjustments

## Data Availability

The RNA sequencing data that support the findings of this study are openly available in the GEO repository, reference number GSE179601.
