# Supplementary material for: Modelling immune cytotoxicity for cholangiocarcinoma with tumour-derived organoids and effector T cells
Source: Br J Cancer. 2022 May 21;127(4):649–60. doi: 10.1038/s41416-022-01839-x (PMC9381772; doi:10.1038/s41416-022-01839-x)
Supplement: Supplementary file 1 — Supplementary Materials [file 41416_2022_1839_MOESM1_ESM.docx]

***Modelling immune cytotoxicity for cholangiocarcinoma with tumor-derived organoids and effector T cells***

**Supplementary Materials**

**Content**

Supplementary Figure S1. Page 2

Supplementary Figure S2. Page 3

Supplementary Figure S3. Page 4

Supplementary Figure S4. Page 5

Supplementary Table S1. Page 6

Supplementary Table S2. Page 7

Supplementary Table S3. Page 8

Supplementary Table S4. Page 9

Supplementary Materials and Methods Page 10

References Page 13

**
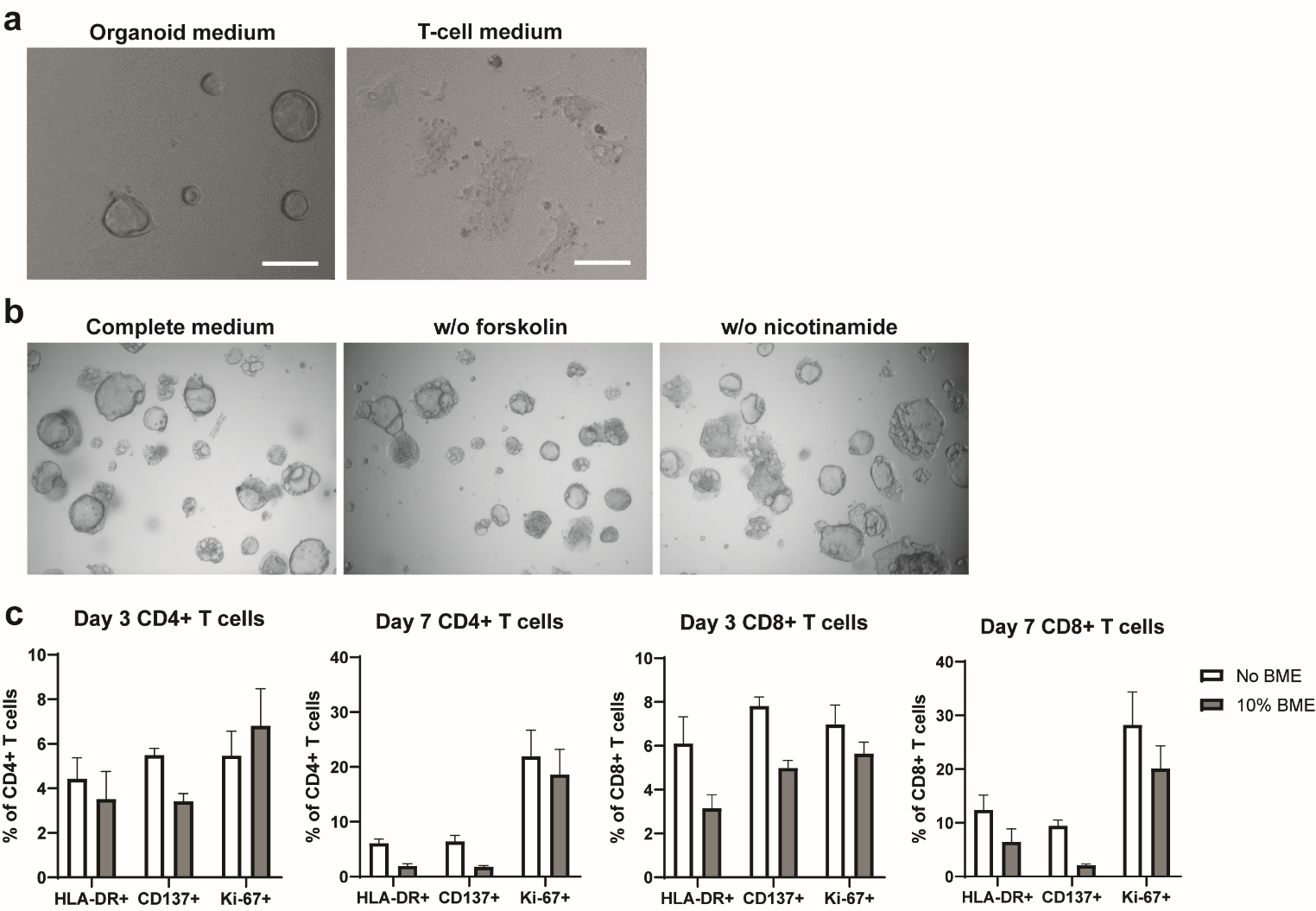
**

**Supplementary Figure S1. Additional evaluation of the effect of different medium compositions on CCA organoids.**

Bright field images demonstrate that CCA organoids cultured in T cell medium (TM) don’t retain the morphology they have in organoid medium (OM) (**a**). CCA organoids were not affected by the removal of either forskolin or nicotinamide from the organoid medium as shown by bright field images (**b**). PBMC were pre-activated for three days, then cultured in OM without nicotinamide with 10% human serum, in the presence or absence of 10% BME. Flow cytometric analysis for HLA-DR, CD137 and Ki-67 expression in CD4^+^ and CD8^+^ T cells at day three and day seven of PBMC culture (n=3 biological replicates from different PBMC donors) (**c**). Scalebar: 100 µm (**a**), 500 µm (**b**). All values with error bars represent mean with SEM.

**Supplementary Figure S2. Effect of organoid medium components on T cells.**


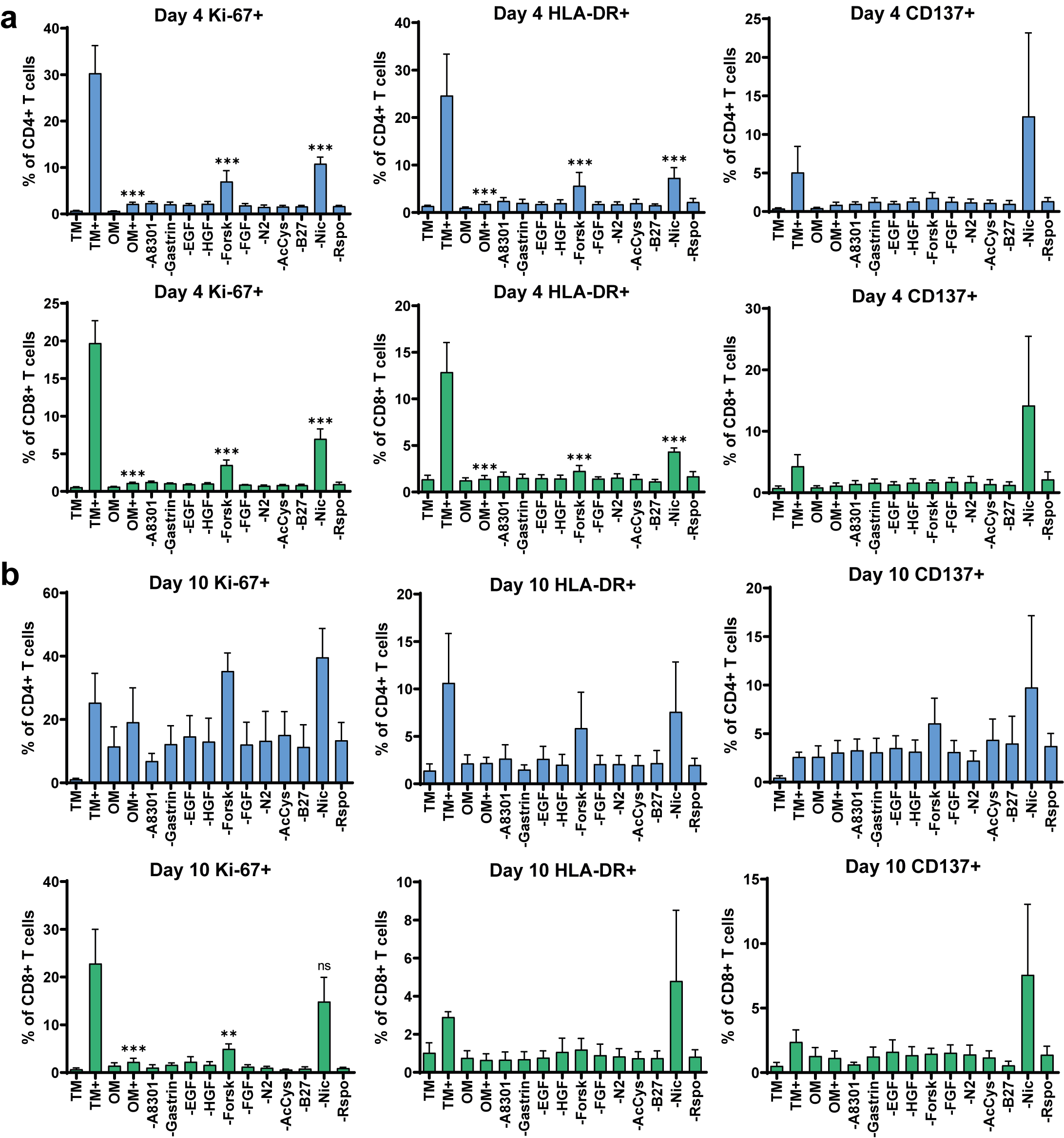


Flow cytometric analysis shown for Ki-67, HLA-DR and CD137 expression in CD4^+^ and CD8^+^ T-cells at day four(**a**) and day ten (**b**) of culture in T cell medium without and with anti-CD3/CD28 coated beads (TM, TM+), organoid medium without and with anti-CD3/CD28 coated beads (OM, OM+), and organoid medium without specified components in the presence of anti-CD3/CD28 coated beads (n=3 biological replicates from different PBMC donors). Statistical significance is depicted for OM+, -Forsk and -Nic compared to TM+. Removal of forskolin or nicotinamide is less effective in reversing the inhibition caused by OM+ compared to TM+ on day four compared to day seven (Fig. 1D). All values with error bars represent mean with SEM. ** p < 0.01, *** p < 0.001.


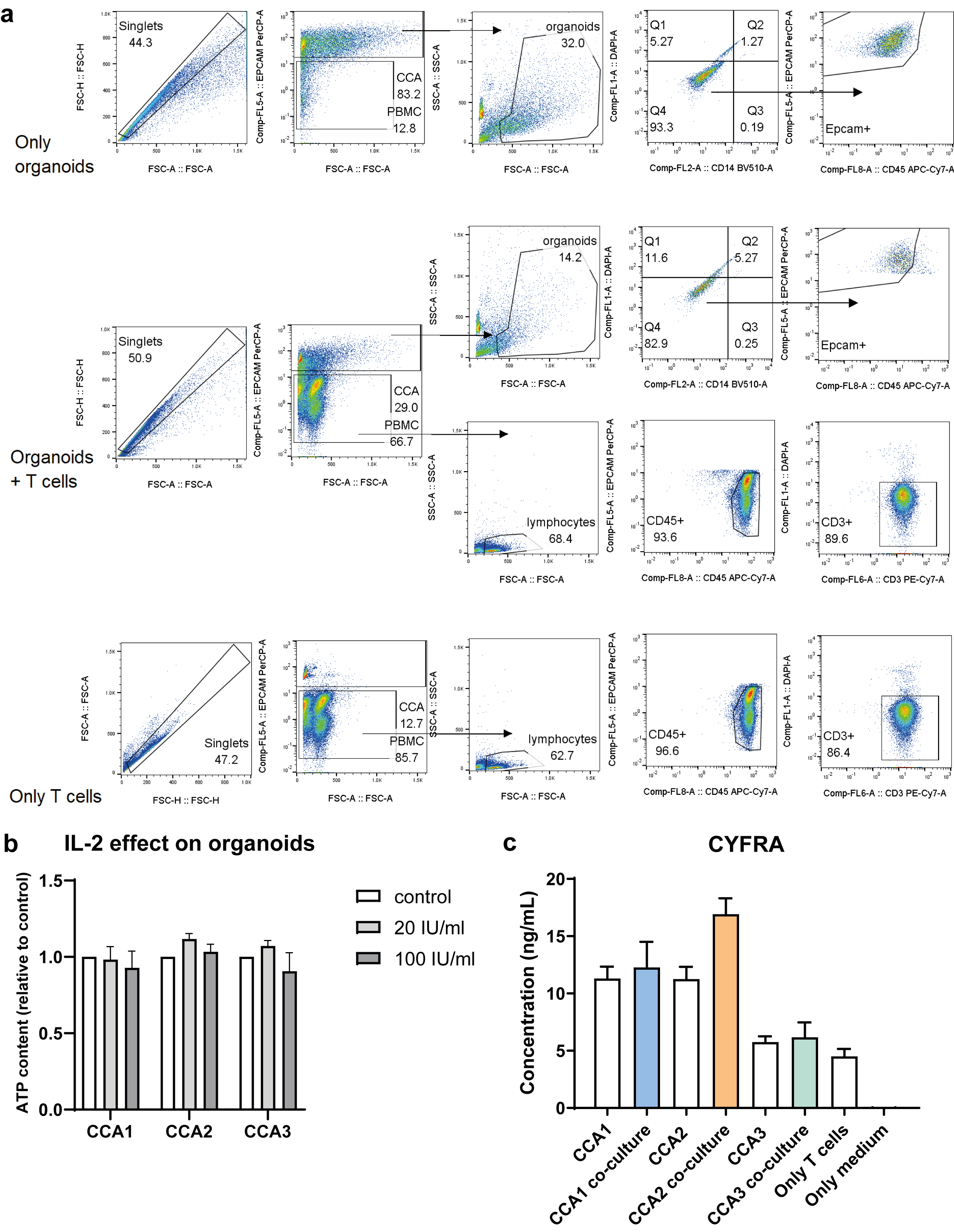


**Supplementary Figure S3. Analysis pipeline for co-culture flow cytometry data and effect of IL-2 on CCA organoid viability.**

Flow cytometry dot plots show the gating strategy of co-culture experiments. Live organoid cells were gated on single cells, EpCAM^+^, DAPI^-^, CD14^-^ and CD45^-^, and live T cells were gated on single cells, EpCAM^-^, CD45^+^, DAPI^-^ and CD3^+^ (**a**). ATP quantification of CCA organoids cultured in organoid medium supplemented with 0, 20 IU/ml (4 ng/ml) or 100 IU/ml (20 ng/ml) IL-2 (n=3 technical replicates) demonstrates IL-2 has no effect on organoid viability (**b**). Quantification of CYFRA in supernatant after seven days of CCA organoid and T cell co-culture (**c**). All values with error bars represent mean with SEM.


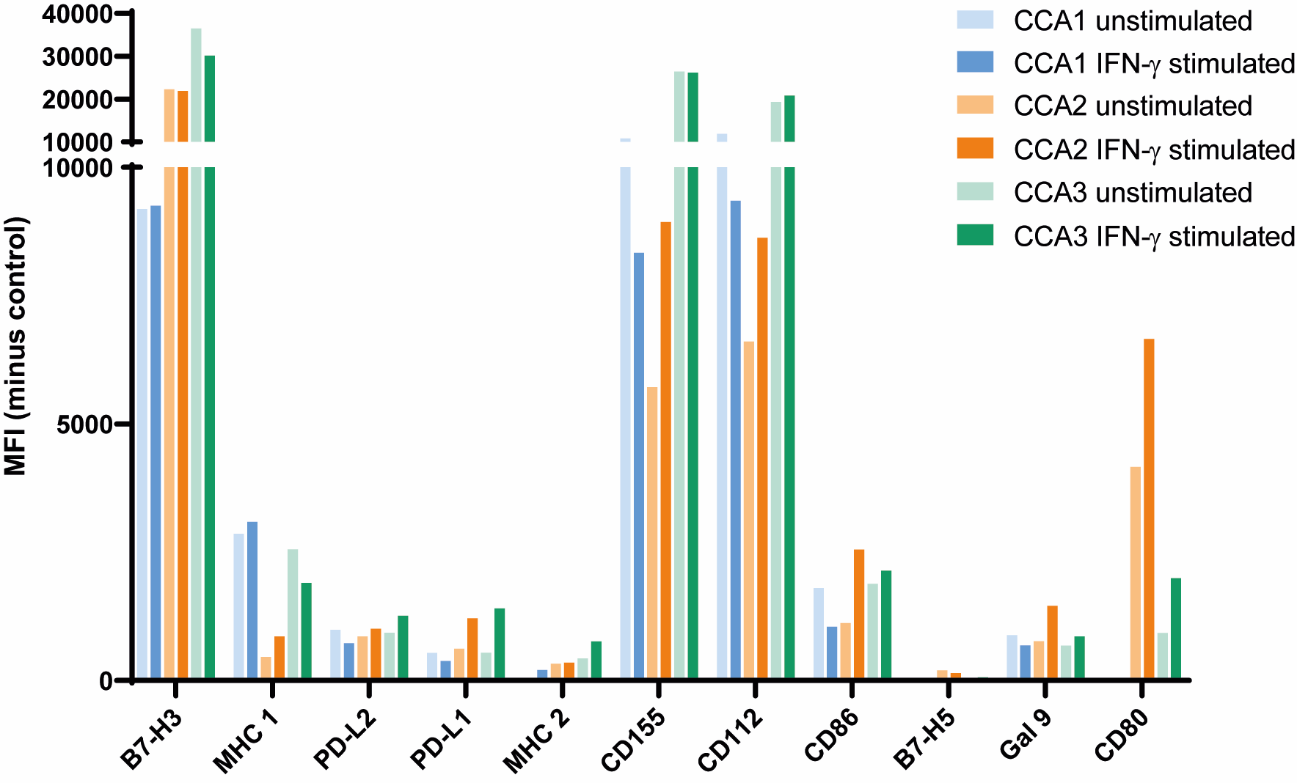


**Supplementary Figure S4. Differences in expression of co-inhibitory and co-stimulatory immune molecules in CCA organoids.**

Flow cytometry for the expression of co-inhibitory and co-stimulatory molecules in CCA organoid lines without and with IFN-γ stimulation. Depicted as median fluorescence intensity (MFI) after subtraction of the control values.

**Supplementary Table S1. Patient characteristics**

|  | **CCA1** | **CCA2** | **CCA3** |
| --- | --- | --- | --- |
| **Type of CCA** | Perihilar | Intrahepatic | Intrahepatic |
| **Sex** | Female | Female | Male |
| **Age** | 34 | 77 | 60 |
| **Pre-treatment** | None | None | None |
| **Etiology** | PSC | Unknown | Unknown |
| **Tumor stage (TNM)** | T2N2M0 | T3N1M0 | T1NXM0 |
| **Tumor differentiation** | Moderate | Moderate to poor | Well to moderate |
| **Fibrosis/Cirrhosis** | No | No | No |
| **Gamma-GT^a^** | 546 | 68 | 93 |
| **AF^a^** | 1831 | 73 | 74 |
| **Bilirubin^a^** | 13 | 8 | 5 |
| **CA19.9^b^** | 42 | 18 | 64 |
| **AFP^b^** | 5 | 4 | - |
| **CEA^b^** | - | 45.8 | - |

^a^Determined 2-4 weeks before surgery. ^b^Determined 4-6 weeks before surgery. Gamma-GT = gamma-glutamyltransferase; AP = alkaline phosphatase; CA19.9 = cancer antigen 19.9; AFP = alpha fetoprotein; CEA = carcinoembryonic antigen.

**Supplementary Table S2. Organoid media compositions**

| **Medium component** | **Manufacturer** | **Organoid initiation medium** | **Organoid expansion medium (OM)** |
| --- | --- | --- | --- |
| Advanced DMEM/F12 | Gibco |  |  |
| HEPES | Life Technologies | 10 mM | 10 mM |
| Ultraglutamine | Lonza | 2 mM | 2 mM |
| Pen/strep | Life Technologies | 100 U/ml | 100 U/ml |
| Primocin | InvivoGen | 0.5 mg/ml | 0.5 mg/ml |
| N2 supplement | Gibco | 1% | 1% |
| B27 supplement (without vitamin A) | Gibco | 2% | 2% |
| Gastrin I | Sigma-Aldrich | 10 nM | 10 nM |
| FGF10 | Peprotech | 100 ng/ml | 100 ng/ml |
| HGF | Peprotech | 25 ng/ml | 25 ng/ml |
| EGF | Peprotech | 50 ng/ml | 50 ng/ml |
| A8301 | Tocris | 5 µM | 5 µM |
| Nicotinamide | Sigma-Aldrich | 10 nM | 10 nM |
| Forskolin | Tocris | 10 µM | 10 µM |
| N-Acetyl Cystein | Sigma-Aldrich | 1 mM | 1 mM |
| R-spondin-1 conditioned medium | Self-produced | 10% | 10% |
| Noggin conditioned medium | Self-produced | 10% |  |
| Wnt3a conditioned medium | Self-produced | 30% |  |
| Y27632 | Tocris | 10 µM |  |
| hES cell cloning recovery solution | Stemgent | 2 µM |  |

**Supplementary Table S3. Anti-human antibodies and reagents used for flow cytometry staining**

| **Antibody / Reagent** | **Clone** | **Isotype** | **Supplier** |
| --- | --- | --- | --- |
| CD56-FITC | TULY56 | mIgG1 | eBioscience-Thermo Fisher Scientific |
| CD8-PE | RPA-T8 | mIgG1, k | Biolegend |
| CD326 (EpCAM)-PerCP-eF710 | 1B7 | mIgG1 | eBioscience-Thermo Fisher Scientific |
| CD45-APC-Fire750 | HI30 | mIgG1,k | Biolegend |
| CD14-BV510 | M5E2 | mIgG2a,k | Biolegend |
| CD3 PE-Cy7 | UCHT1 | mlgG1,k | eBioscience-Thermo Fisher Scientific |
| CD45-APC-eFluor780 | HI30 | mIgG1,k | eBioscience-Thermo Fisher Scientific |
| CD8a-PE | OKT8 | mIgG2a | eBioscience-Thermo Fisher Scientific |
| CD4-APC-eFluor780 | OKT4 | mIgG2b,k | eBioscience-Thermo Fisher Scientific |
| CD45-APC | HI30 | mIgG1,k | Biolegend |
| CD4-FITC | Oct-04 | mIgG2b,k | Biolegend |
| CD4 PE | 13B8.2 | mlgG1 | Beckman |
| HLA-DR-PerCP-Cy5.5 | LN3 | mlgG2b | eBioscience-Thermo Fisher Scientific |
| CD137 (4-1BB)-APC | 4B4-1 | mIgG1,k | BD Pharmingen |
| CD8-efluor450 | RPA-T8 | mIgG1,k | eBioscience-Thermo Fisher Scientific |
| CD45-APC-eFluor780 | HI30 | mIgG1,k | eBioscience-Thermo Fisher Scientific |
| Ki67-FITC | B56 | mIgG1,k | BD pharmingen |
| CD4 PerCP-Cy5.5 | SK3 | mlgG1 | B&D |
| HLA-DR-PE | LN3 | mIgG2b | eBioscience-Thermo Fisher Scientific |
| Ki67-FITC | 20Raj1 | mIgG1,k | eBioscience-Thermo Fisher Scientific |
| CD273 (B7-DC)-PE | MIH18 | mIgG1 | eBioscience-Thermo Fisher Scientific |
| HLA-A,B,C-FITC | W6/32 | mIgG2a,k | Biolegend |
| CD274(B7-H1)-PE-Cy7 | MIH1 | mIgG1 | eBioscience-Thermo Fisher Scientific |
| CD276(B7-H3)-APC | 7-517 | mIgG1,k | eBioscience-Thermo Fisher Scientific |
| HLA-DR, DP, DQ-FITC | Tü39 | miGG2a,k | Biolegend |
| CD155-PE | 2H7CD155 | mIgG1 | eBioscience-Thermo Fisher Scientific |
| CD112-PECy7 | TX31 | mIgG1 | Biolegend |
| CD274 (B7-H1)-APC | MIH1 | mIgG1 | eBioscience-Thermo Fisher Scientific |
| Vista(B7-H5)-AlexaFluor488 | #730804 | mIgG2b | R&D systems |
| Galectin-9 PE | 9M1-3 | mIgG1,k | Biolegend |
| CD80-PE-Cy7 | 2D10 | mgG1,k | Biolegend |
| CD86 APC | IT2.2 | mlgG2b,k | Biolegend |
| IFN-gamma-APC-R700 | B27 | mIgG1,k | BD Biosciences |
| TNFa-FITC | MAb11 | mIgG1,k | eBioscience-Thermo Fisher Scientific |
| Human Fc block |  |  | BD Biosciences |
| Annexin V apoptosis detection kit APC |  |  | eBioscience -Thermo Fisher Scientific |
| DAPI |  |  | Biolegend |
| Fixable viability dye eFluor 506 |  |  | eBioscience-Thermo Fisher Scientific |

**Supplementary Table S4. Genes identified in the set of 1000 upregulated genes in CCA1 that are part of the cell adhesion-related biological processes**

| **GO:0007156** | **GO:0007155** | **GO:0016339** |
| --- | --- | --- |
| RET | FBLN7 | NLGN1 |
| PCDHGB7 | SRPX | PCDHGB4 |
| TENM3 | NLGN1 | PCDHB16 |
| PCDHGB5 | COL16A1 | PCDHB6 |
| PCDHGB4 | PCDHGB4 | PCDHB5 |
| PCDH10 | PCDH10 | PCDHB13 |
| PCDH17 | TGFB1I1 | PCDHB3 |
| ROBO1 | ITGB2 | CDH16 |
| CDH7 | NID2 | PCDHB11 |
| PCDHA1 | THBS4 |  |
| PCDHA3 | PCDH17 |  |
| PCDHA2 | ROBO1 |  |
| PCDH9 | PCDHA1 |  |
| PCDHGA3 | CHST10 |  |
| PCDHGA2 | PCDHA3 |  |
| PCDH20 | CTNNA3 |  |
| PCDHGA1 | PCDHA2 |  |
| PCDHB15 | MPDZ |  |
| PCDHB13 | EDIL3 |  |
| PCDHB11 | IL32 |  |
| PCDHGA10 | MYBPC1 |  |
| PCDHGA11 | CD72 |  |
| PCDHGA12 | AMBP |  |
| PCDHB16 | PCDHB15 |  |
| PCDHB6 | PCDHB11 |  |
| PCDHB5 | CLDN10 |  |
| PCDHB3 | BCAM |  |
| FAT4 | MMRN1 |  |
| CDH16 | PTK7 |  |
| PCDHB8 | ADGRB1 |  |
|  | PCDHB6 |  |
|  | CASS4 |  |
|  | CERCAM |  |
|  | ANOS1 |  |
|  | CNTN1 |  |
|  | CNTN3 |  |
|  | SPACA4 |  |
|  | PCDHB3 |  |
|  | ITGA5 |  |
|  | CDH16 |  |
|  | EPHA3 |  |
|  | ITGA9 |  |

**Supplementary Materials and Methods**

***Organoid culture***

In short, biopsies were washed in Advanced DMEM/F12 (Gibco), minced, and digested using a 2.5 mg/ml collagenase A solution (Sigma Aldrich). The cell suspension was strained through a 70 µm strainer, cells were washed in Advanced DMEM/F12 and plated in 75% BME (Cultrex, diluted in Advanced DMEM/F12). Until splitting, organoids were maintained in classical organoid initiation medium as described by Huch et al.^1^. After the first split, culture was continued in organoid expansion medium (OM) which was refreshed twice a week. Organoids were passaged in 1:3 to 1:10 split ratios once a week. Exact medium composition can be found in Supplementary Table S2.

***Co-culture assay for immune cell analysis***

PBMC were thawed 2-3 days in advance at 37°C, then resuspended and cultured in TM either with or without anti-human CD3/CD28 dynabeads at a 1:20 bead:cell ratio, in the presence of 20 IU/ml (4 ng/ml) IL-2. On the day of co-culture, organoids were harvested, mechanically broken, and dissociated into single cells by TrypLE Express incubation (3-5 cycles of 5 minutes at 37°C). PBMC were harvested, counted, and either cultured alone or combined with organoid single cells at a 50000:25000 = 2:1 ratio. They were resuspended in 200 µL OM-nic with 10% human serum and plated in a 96-well round-bottom plate. 100 IU/ml (20 ng/ml) IL-2 was added to some conditions, and 5 µg/mL brefeldin (Sigma) and 1:1000 monensin (eBioscience Invitrogen) were added to all conditions. After 19 hours, cells were washed, stained with fixable viability dye eFluor 506 first and then anti-EpCAM, anti-CD45, anti-CD3, anti-CD8 and anti-CD4, followed by intra-cellular staining of IFN-γ and TNF-α using the Foxp3 staining buffer set.

***Effect of BME on T cells***

PBMC from healthy donors were thawed at 37°C, then resuspended and cultured in TM with anti-human CD3/CD28 dynabeads at a 1:100 bead:cell ratio, in the presence of 20 IU/ml (4 ng/ml) IL-2. After three days, PBMC were harvested, counted and resuspended in 200 µL OM-nic with 10% human serum, in the presence or absence of 10% BME. They were plated in a 96-well flat-bottom suspension plate. After three and seven days, respectively, PBMC were harvested and stained with fixable viability dye eFluor 506. Cell surface staining was then performed with anti-CD45, anti-CD8, anti-CD4, anti-CD3, anti-HLA-DR and anti-CD137 antibodies (Suppl. Table S3). For Ki-67 staining, cells were fixed and permeabilized using the Foxp3 staining buffer set. PBMC were measured using a FACSCanto II flow cytometer and analyzed using FlowJo software.

***Confocal time-lapse imaging settings and analysis***

The blue nuclear Hoechst 33342 staining, green caspase 3/7 probe and CellTrace Far Red were sequentially imaged using 405, 488 and 640 nm solid state lasers for excitation and detected at 435-480, 500-550 and 650-760 nm, respectively. An additional bright field channel was included. For 180 hours, every 6 hours 9 z-stacks of 7 planes with 7.4 µm interval were imaged covering 9 times 1284550 µm2 and 44.4 µm in height.

Maximum projections of the z-stacks were analyzed with a custom analysis pipeline in the Opera Phenix Harmony PhenoLOGIC software (Perkin Elmer). In short, organoids were segmented based on the combined Hoechst and green caspase signals. Within the segmented organoid area, the percentage surface area of dead cells was determined using the green caspase 3/7 probe channel, reflecting the level of cell death within the individual organoids. T cells were detected using the far-red signal.

***TIL isolation***

Single cell suspensions from tumors and tumor-free liver tissues were obtained by tissue digestion as described previously^2^. Briefly, fresh tissues were first cut into small pieces, and then digested with 0.125 mg/mL collagenase IV (Sigma-Aldrich), 0.2 mg/mL DNAse I (Roche,), 1000 IU/ml hyaluronidase (Sigma), and 0.05% FCS in [Hanks' Balanced Salt solution](http://www.sigmaaldrich.com/catalog/product/sigma/h6648?lang=en&region=US) with Ca^2+^ and Mg^2+^ (Sigma) for 30-60 minutes at 37 °C with continuous stirring. Cell suspensions were filtered through 100 µm pore cell strainers (BD Biosciences) and mononuclear immune cells were obtained by Ficoll density gradient centrifugation. Viability, cell count and purity were determined by trypan blue exclusion and MacsQuant (Miltenyi).

***Preparation of conditioned media from PBMC and TIL***

PBMC from healthy donors or TIL from CCA or hepatocellular carcinoma patients were thawed at 37°C, then resuspended and cultured in the presence or absence of anti-human CD3/CD28 dynabeads at a 1:100 bead:cell ratio at a concentration of 0.5-1 x 10^6^ cells/ml OM-nic with 10% human serum. OM-nic with 10% human serum without cells was used as control medium. Conditioned medium was collected on day six and stored at 4°C until the next day or frozen at -20°C until later use. On the day of use, the conditioned medium and control medium were concentrated 10-fold by centrifugation at 3100-3200 g for 30-40 minutes at 20°C using 3KDa centrifugal filters (Amicon Ultra 3K device-3000MWCO).

***RNA sequencing***

RNA from three CCA organoid lines was collected in Qiazol lysis buffer (Qiagen) and isolated using the miRNeasy mini kit (Qiagen) according to manufacturer’s instructions. Subsequently, RNA sequencing was performed by Novogene, which resulted in approximately 20-30 million, paired-end reads. The sequencing data were uploaded to the Galaxy Web platform public server usegalaxy.org^3^. Next, the data were trimmed for adapters using Trim Galore! (version 0.4.3.1), and mapped using RNA STAR (version 2.6.8a) against the human reference genome GRCh38. By applying the built-in hg38 genome annotation file, mapped reads were translated into raw counts using FeatureCounts (version 2.0.1).

**References**

1. Huch M, Gehart H, van Boxtel R, Hamer K, Blokzijl F, Verstegen MM, et al. Long-term culture of genome-stable bipotent stem cells from adult human liver. *Cell* **160**, 299-312 (2015).

2. Zhou G, Sprengers D, Mancham S, Erkens R, Boor PPC, van Beek AA, et al. Reduction of immunosuppressive tumor microenvironment in cholangiocarcinoma by ex vivo targeting immune checkpoint molecules. *J Hepatol* **71**, 753-62 (2019).

3. Afgan E, Baker D, van den Beek M, Blankenberg D, Bouvier D, Cech M, et al. The Galaxy platform for accessible, reproducible and collaborative biomedical analyses: 2016 update. *Nucleic Acids Res* **44**, W3-W10 (2016).
