# Supplementary material for: Modelling immune cytotoxicity for cholangiocarcinoma with tumour-derived organoids and effector T cells
Source: Br J Cancer. 2022 May 21;127(4):649–60. doi: 10.1038/s41416-022-01839-x (PMC9381772; doi:10.1038/s41416-022-01839-x)
Supplement: Supplementary file 6 — Approval of author list adjustments [file 41416_2022_1839_MOESM6_ESM.pdf]

Monique Verstegen

From: Monique Verstegen
Sent: Tuesday, April 19, 2022 5:48 PM
To: Guoying Zhou; Ruby Lieshout; Gilles van Tienderen; valeska@glycostem.com; Martin van Royen; Kübra Koten; y.y.kan@amsterdamumc.nl; 'Cecile Geuijen <C.Geuijen@merus.nl> (C.Geuijen@merus.nl)' (C.Geuijen@merus.nl); Dave Sprengers; Luc van der Laan; Monique Verstegen; luciacampos3190@gmail.com; gezhouhong37@hotmail.com
Cc: Maikel Peppelenbosch; magreluc; Patrick Boor; Jyaysi Desai
Subject: Important! Please respond asap / acceptance manuscript BJC

Importance: High

Follow Up Flag: Follow up
Flag Status: Flagged

Dear co-authors,

We are nearly there! Manuscript: BJC-A3339175R1 Modelling immune cytotoxicity for cholangiocarcinoma with tumor-derived organoids and effector T cells., gave us a small hick up that is fortunately easily addressed.

As we needed to do more experiments in absence of Ruby and Estella, we asked Luc (M), Patrick and Jyaysi if they could help us. And they did! So, all were included as co-authors on this manuscript. The Journal now would like to formally ask all of you if you agree to those changes. For this I would need an e-mail from you, in which you confirm that you agree.

Can you please send me your reply to this e-mail, including a statement that you agree to the changes made, i.e. including Luc Magre, Patrick P.C. Boor and Jyaysi Desai, in the author list at you soonest so I can bundle all replies and upload them to BJC.

The BJC also wanted me to contact you to ask you to link your own ORCID to this manuscript. You can do this via the author portal here: https://mts-bjcancer.nature.com/cgi-bin/main.plex and click on the 'Modify My Springer Nature Account' (see below)

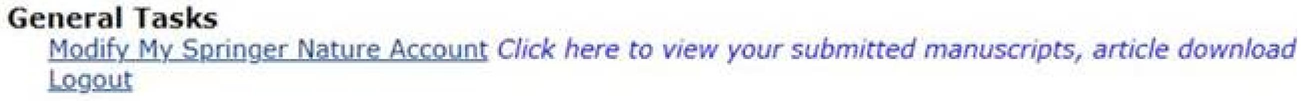

Hope to see your reply soon,

With kind regards,
Monique Verstegen
Corresponding author Manuscript: BJC-A3339175R1

Dr. M.M.A (Monique) Verstegen Ph.D

Assistant professor

Surgery

+31 10 703 5528 | Dr. Molewaterplein 40, 3015 GD Rotterdam

m.verstegen@erasmusmc.nl
www.erasmusmc.nl

P.O. Box 2040, 3000 CA Rotterdam

Monique Verstegen

From: Cecile Geuijen <C.Geuijen@merus.nl>
Sent: Tuesday, April 19, 2022 9:42 PM
To: Monique Verstegen
Subject: RE: Important! Please respond asap / acceptance manuscript BJC

Follow Up Flag: Flag for follow up
Flag Status: Flagged

Waarschuwing: Deze e-mail is afkomstig van buiten de organisatie. Klik niet op links en open geen bijlagen, tenzij u de afzender herkent en weet dat de inhoud veilig is.
Caution: This email originated from outside of the organization. Do not click links or open attachments unless you recognize the sender and know the content is safe.

Hi Monique,

Herewith I agree to the changes made, and to include Luc Magre, Patrick P.C. Boor and Jyaysi Desai to the author list for providing the latest data.

Met vriendelijke groet / kind regards,

Cecile Geuijen
Chief Scientific Officer

T +31 85 016 2558
M +31 6 15 54 23 83
E C.Geuijen@merus.nl

Merus closing in on cancer

From: Monique Verstegen <m.verstegen@erasmusmc.nl>
Sent: Tuesday, April 19, 2022 5:48 PM
To: Guoying Zhou <g.zhou@erasmusmc.nl>, Ruby Lieshout <r.lieshout@erasmusmc.nl>, Gilles van Tienderen <g.vantienderen@erasmusmc.nl>, valeska@glycostem.com <valeska@glycostem.com>, Martin van Royen <m.vanroyen@erasmusmc.nl>,; Kübra Koten
Cc: m.peppelenbosch <m.peppelenbosch@erasmusmc.nl>; magreluc <magreluc@gmail.com>; Patrick Boor <p.boor@erasmusmc.nl>; J.B. Desai <j.desai@erasmusmc.nl>
Subject: Important! Please respond asap / acceptance manuscript BJC

External Mail

Dear co-authors,

We are nearly there! Manuscript: BJC-A3339175R1 Modelling immune cytotoxicity for cholangiocarcinoma with tumor-derived organoids and effector T cells., gave us a small hick up that is fortunately easily addressed.

As we needed to do more experiments in absence of Ruby and Estella, we asked Luc (M), Patrick and Jyaysi if they could help us. And they did! So, all were included as co-authors on this manuscript. The Journal now would like to formally ask all of you if you agree to those changes. For this I would need an e-mail from you, in which you confirm that you agree.

<u>Can you please send me your reply to this e-mail, including a statement that you agree to the changes made, i.e. including Luc Magre, Patrick P.C. Boor and Jyaysi Desai, in the author list at you soonest</u> so I can bundle all replies and upload them to BJC.

The BJC also wanted me to contact you to ask you to link your own ORCID to this manuscript. You can do this via the author portal here: nature.com and click on the ‘Modify My Springer Nature Account’ (see below)

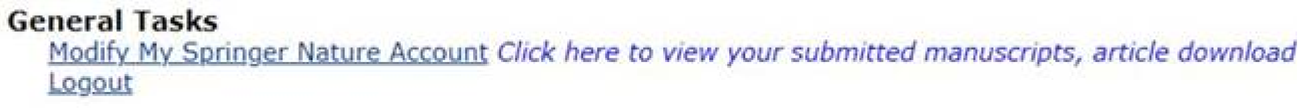

Hope to see your reply soon,

With kind regards,
Monique Verstegen
Corresponding author Manuscript: BJC-A3339175R1

Dr. M.M.A (Monique) Verstegen Ph.D

Assistant professor

Surgery

+31 10 703 5528 |
m.verstegen@erasmusmc.nl
www.erasmusmc.nl

Dr. Molewaterplein 40, 3015 GD Rotterdam
P.O. Box 2040, 3000 CA Rotterdam

Monique Verstegen

**From:**	Dave Sprengers
**Sent:**	Tuesday, April 19, 2022 6:28 PM
**To:**	Monique Verstegen
**Subject:**	RE: Important! Please respond asap / acceptance manuscript BJC
**Follow Up Flag:**	Flag for follow up
**Flag Status:**	Flagged

Dear Monique,

I agree to the changes made, i.e. including Luc Magre, Patrick P.C. Boor and Jyaysi Desai, in the author list.

Regards,

Dave Sprengers

MDL-arts
Erasmus MC

Van: Monique Verstegen <m.verstegen@erasmusmc.nl>
Verzonden: dinsdag 19 april 2022 17:48
Aan: Guoying Zhou <g.zhou@erasmusmc.nl>; Ruby Lieshout <r.lieshout@erasmusmc.nl>; Gilles van Tienderen <g.vantienderen@erasmusmc.nl>; valeska@glycostem.com; Martin van Royen <m.vanroyen@erasmusmc.nl>; Kübra Koten <k.koten@erasmusmc.nl>; y.y.kan@amsterdamumc.nl; 'Cecile Geuijen <C.Geuijen@merus.nl> (C.Geuijen@merus.nl)' (C.Geuijen@merus.nl) <C.Geuijen@merus.nl>; Dave Sprengers <d.sprengers@erasmusmc.nl>; Luc van der Laan <l.vanderlaan@erasmusmc.nl>; Monique Verstegen <m.verstegen@erasmusmc.nl>; luciacampos3190@gmail.com; gezhouhong37@hotmail.com
CC: Maikel Peppelenbosch <m.peppelenbosch@erasmusmc.nl>; magreluc <magreluc@gmail.com>; Patrick Boor <p.boor@erasmusmc.nl>; Jyaysi Desai <j.desai@erasmusmc.nl>
Onderwerp: Important! Please respond asap / acceptance manuscript BJC
Urgentie: Hoog

Dear co-authors,

We are nearly there! Manuscript: BJC-A3339175R1 Modelling immune cytotoxicity for cholangiocarcinoma with tumor-derived organoids and effector T cells., gave us a small hick up that is fortunately easily addressed.

As we needed to do more experiments in absence of Ruby and Estella, we asked Luc (M), Patrick and Jyaysi if they could help us. And they did! So, all were included as co-authors on this manuscript. The Journal now would like to formally ask all of you if you agree to those changes. For this I would need an e-mail from you, in which you confirm that you agree.

Can you please send me your reply to this e-mail, including a statement that you agree to the changes made, i.e. including Luc Magre, Patrick P.C. Boor and Jyaysi Desai, in the author list at you soonest so I can bundle all replies and upload them to BJC.

The BJC also wanted me to contact you to ask you to link your own ORCID to this manuscript. You can do this via the author portal here: https://mts-bjcancer.nature.com/cgi-bin/main.plex and click on the 'Modify My Springer Nature Account' (see below)

General Tasks
Modify My Springer Nature Account *Click here to view your submitted manuscripts, article download*
Logout

Hope to see your reply soon,

With kind regards,
Monique Verstegen
Corresponding author Manuscript: BJC-A3339175R1

Dr. M.M.A (Monique) Verstegen Ph.D

Assistant professor

Surgery

+31 10 703 5528 |
m.verstegen@erasmusmc.nl
www.erasmusmc.nl

Dr. Molewaterplein 40, 3015 GD Rotterdam
P.O. Box 2040, 3000 CA Rotterdam

Monique Verstegen

From: Gilles van Tienderen
Sent: Wednesday, April 20, 2022 11:55 AM
To: Monique Verstegen
Subject: Re: Important! Please respond asap / acceptance manuscript BJC

Dear Monique,

I agree to the changes made in the manuscript BJC-A3339175R1

Kind regards,

Gilles van Tienderen

From: Monique Verstegen <m.verstegen@erasmusmc.nl>
Sent: Tuesday, April 19, 2022 4:48:12 PM
To: Guoying Zhou <g.zhou@erasmusmc.nl>; Ruby Lieshout <r.lieshout@erasmusmc.nl>; Gilles van Tienderen <g.vantienderen@erasmusmc.nl>; valeska@glycostem.com <valeska@glycostem.com>; Martin van Royen <m.vanroyen@erasmusmc.nl>; Kübra Koten <k.koten@erasmusmc.nl>; y.y.kan@amsterdamumc.nl <y.y.kan@amsterdamumc.nl>; 'Cecile Geuijen <C.Geuijen@merus.nl> (C.Geuijen@merus.nl)' (C.Geuijen@merus.nl) <C.Geuijen@merus.nl>; Dave Sprengers <d.sprengers@erasmusmc.nl>; Luc van der Laan <l.vanderlaan@erasmusmc.nl>; Monique Verstegen <m.verstegen@erasmusmc.nl>; luciacampos3190@gmail.com <luciacampos3190@gmail.com>; gezhouhong37@hotmail.com <gezhouhong37@hotmail.com>
Cc: Maikel Peppelenbosch <m.peppelenbosch@erasmusmc.nl>; magreluc <magreluc@gmail.com>; Patrick Boor <p.boor@erasmusmc.nl>; Jyaysi Desai <j.desai@erasmusmc.nl>
Subject: Important! Please respond asap / acceptance manuscript BJC

Dear co-authors,

We are nearly there! Manuscript: BJC-A3339175R1 Modelling immune cytotoxicity for cholangiocarcinoma with tumor-derived organoids and effector T cells., gave us a small hick up that is fortunately easily addressed.

As we needed to do more experiments in absence of Ruby and Estella, we asked Luc (M), Patrick and Jyaysi if they could help us. And they did! So, all were included as co-authors on this manuscript. The Journal now would like to formally ask all of you if you agree to those changes. For this I would need an e-mail from you, in which you confirm that you agree.

Can you please send me your reply to this e-mail, including a statement that you agree to the changes made, i.e. including Luc Magre, Patrick P.C. Boor and Jyaysi Desai, in the author list at you soonest so I can bundle all replies and upload them to BJC.

The BJC also wanted me to contact you to ask you to link your own ORCID to this manuscript. You can do this via the author portal here: https://mts-bjcancer.nature.com/cgi-bin/main.plex and click on the 'Modify My Springer Nature Account' (see below)

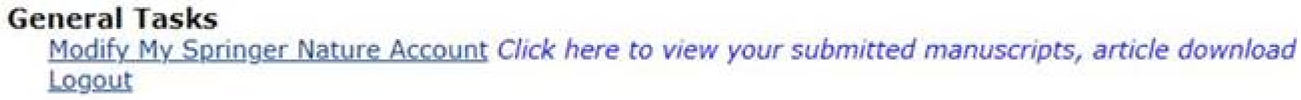

Hope to see your reply soon,

With kind regards,
Monique Verstegen
Corresponding author Manuscript: BJC-A3339175R1

Dr. M.M.A (Monique) Verstegen Ph.D

Assistant professor

Surgery

+31 10 703 5528 |
m.verstegen@erasmusmc.nl
www.erasmusmc.nl

Dr. Molewaterplein 40, 3015 GD Rotterdam
P.O. Box 2040, 3000 CA Rotterdam

Monique Verstegen

From: Guoying Zhou
Sent: Wednesday, April 20, 2022 8:38 AM
To: Monique Verstegen
Subject: Re: Important! Please respond asap / acceptance manuscript BJC

Follow Up Flag: Follow up
Flag Status: Completed

I agree to the changes made, i.e. including Luc Magre, Patrick P.C. Boor and Jyaysi Desai, in the author list.
Best regards,
Guoying Zhou

From: Monique Verstegen <m.verstegen@erasmusmc.nl>
Sent: Tuesday, April 19, 2022 11:48:12 PM
To: Guoying Zhou <g.zhou@erasmusmc.nl>; Ruby Lieshout <r.lieshout@erasmusmc.nl>; Gilles van Tienderen <g.vantienderen@erasmusmc.nl>; valeska@glycostem.com <valeska@glycostem.com>; Martin van Royen <m.vanroyen@erasmusmc.nl>; Kübra Koten <k.koten@erasmusmc.nl>; y.y.kan@amsterdamumc.nl <y.y.kan@amsterdamumc.nl>; 'Cecile Geuijen <C.Geuijen@merus.nl> (C.Geuijen@merus.nl)' (C.Geuijen@merus.nl) <C.Geuijen@merus.nl>; Dave Sprengers <d.sprengers@erasmusmc.nl>; Luc van der Laan <l.vanderlaan@erasmusmc.nl>; Monique Verstegen <m.verstegen@erasmusmc.nl>; luciacampos3190@gmail.com <luciacampos3190@gmail.com>; gezhouhong37@hotmail.com <gezhouhong37@hotmail.com>
Cc: Maikel Peppelenbosch <m.peppelenbosch@erasmusmc.nl>; magreluc <magreluc@gmail.com>; Patrick Boor <p.boor@erasmusmc.nl>; Jyaysi Desai <j.desai@erasmusmc.nl>
Subject: Important! Please respond asap / acceptance manuscript BJC

Dear co-authors,

We are nearly there! Manuscript: BJC-A3339175R1 Modelling immune cytotoxicity for cholangiocarcinoma with tumor-derived organoids and effector T cells., gave us a small hick up that is fortunately easily addressed.

As we needed to do more experiments in absence of Ruby and Estella, we asked Luc (M), Patrick and Jyaysi if they could help us. And they did! So, all were included as co-authors on this manuscript. The Journal now would like to formally ask all of you if you agree to those changes. For this I would need an e-mail from you, in which you confirm that you agree.

Can you please send me your reply to this e-mail, including a statement that you agree to the changes made, i.e. including Luc Magre, Patrick P.C. Boor and Jyaysi Desai, in the author list at you soonest so I can bundle all replies and upload them to BJC.

The BJC also wanted me to contact you to ask you to link your own ORCID to this manuscript. You can do this via the author portal here: https://mts-bjcancer.nature.com/cgi-bin/main.plex and click on the ‘Modify My Springer Nature Account’ (see below)

Hope to see your reply soon,

With kind regards,
Monique Verstegen

Corresponding author Manuscript: BJC-A3339175R1

Dr. M.M.A (Monique) Verstegen Ph.D

Assistant professor

Surgery

+31 10 703 5528 |
m.verstegen@erasmusmc.nl
www.erasmusmc.nl

Dr. Molewaterplein 40, 3015 GD Rotterdam
P.O. Box 2040, 3000 CA Rotterdam

The linked image cannot be displayed. The file may have been moved, renamed, or deleted. Verify that the link points to the correct file and location.

The linked image cannot be displayed. The file may have been moved, renamed, or deleted. Verify that the link points to the correct file and location.

Monique Verstegen

**From:**	Jyaysi Desai
**Sent:**	Wednesday, April 20, 2022 10:22 AM
**To:**	Monique Verstegen; Guoying Zhou; Ruby Lieshout; Gilles van Tienderen; valeska@glycostem.com; Martin van Royen; Kübra Koten; y.y.kan@amsterdamumc.nl; 'Cecile Geuijen <C.Geuijen@merus.nl> (C.Geuijen@merus.nl)' (C.Geuijen@merus.nl); Dave Sprengers; Luc van der Laan; luciacampos3190@gmail.com; gezhouhong37@hotmail.com
**Cc:**	Maikel Peppelenbosch; magreluc; Patrick Boor
**Subject:**	RE: Important! Please respond asap / acceptance manuscript BJC

Dear all,

I agree to the changes made in the manuscript BJC-A3339175R1.

Kind regards,
Jyaysi

From: Monique Verstegen <m.verstegen@erasmusmc.nl>
Sent: Tuesday, April 19, 2022 5:48 PM
To: Guoying Zhou <g.zhou@erasmusmc.nl>; Ruby Lieshout <r.lieshout@erasmusmc.nl>; Gilles van Tienderen <g.vantienderen@erasmusmc.nl>; valeska@glycostem.com; Martin van Royen <m.vanroyen@erasmusmc.nl>; Kübra Koten <k.koten@erasmusmc.nl>; y.y.kan@amsterdamumc.nl; 'Cecile Geuijen <C.Geuijen@merus.nl> (C.Geuijen@merus.nl)' (C.Geuijen@merus.nl) <C.Geuijen@merus.nl>; Dave Sprengers <d.sprengers@erasmusmc.nl>; Luc van der Laan <l.vanderlaan@erasmusmc.nl>; Monique Verstegen <m.verstegen@erasmusmc.nl>; luciacampos3190@gmail.com; gezhouhong37@hotmail.com
Cc: Maikel Peppelenbosch <m.peppelenbosch@erasmusmc.nl>; magreluc <magreluc@gmail.com>; Patrick Boor <p.boor@erasmusmc.nl>; Jyaysi Desai <j.desai@erasmusmc.nl>
Subject: Important! Please respond asap / acceptance manuscript BJC
Importance: High

Dear co-authors,

We are nearly there! Manuscript: BJC-A3339175R1 Modelling immune cytotoxicity for cholangiocarcinoma with tumor-derived organoids and effector T cells., gave us a small hick up that is fortunately easily addressed.

As we needed to do more experiments in absence of Ruby and Estella, we asked Luc (M), Patrick and Jyaysi if they could help us. And they did! So, all were included as co-authors on this manuscript. The Journal now would like to formally ask all of you if you agree to those changes. For this I would need an e-mail from you, in which you confirm that you agree.

Can you please send me your reply to this e-mail, including a statement that you agree to the changes made, i.e. including Luc Magre, Patrick P.C. Boor and Jyaysi Desai, in the author list at you soonest so I can bundle all replies and upload them to BJC.

The BJC also wanted me to contact you to ask you to link your own ORCID to this manuscript. You can do this via the author portal here: https://mts-bjcancer.nature.com/cgi-bin/main.plex and click on the 'Modify My Springer Nature Account' (see below)

General Tasks

Modify My Springer Nature Account *Click here to view your submitted manuscripts, article download*

Logout

Hope to see your reply soon,

With kind regards,
Monique Verstegen
Corresponding author Manuscript: BJC-A3339175R1

Dr. M.M.A (Monique) Verstegen Ph.D

Assistant professor

Surgery

+31 10 703 5528 |
m.verstegen@erasmusmc.nl
www.erasmusmc.nl

Dr. Molewaterplein 40, 3015 GD Rotterdam
P.O. Box 2040, 3000 CA Rotterdam

Erasmus MC
TRANSPLANT INSTITUTE

Monique Verstegen

From: Kübra Koten
Sent: Wednesday, April 20, 2022 10:45 AM
To: Jyaysi Desai; Monique Verstegen; Guoying Zhou; Ruby Lieshout; Gilles van Tienderen; valeska@glycostem.com; Martin van Royen; y.y.kan@amsterdamumc.nl; 'Cecile Geuijen <C.Geuijen@merus.nl> (C.Geuijen@merus.nl)' (C.Geuijen@merus.nl); Dave Sprengers; Luc van der Laan; luciacampos3190@gmail.com; gezhouhong37@hotmail.com
Cc: Maikel Peppelenbosch; magreluc; Patrick Boor
Subject: RE: Important! Please respond asap / acceptance manuscript BJC

Dear all,

I agree to the changes made in the manuscript BJC-A3339175R1.

Kind regards,

Kübra Köten

From: Jyaysi Desai <j.desai@erasmusmc.nl>
Sent: Wednesday, April 20, 2022 10:22
To: Monique Verstegen <m.verstegen@erasmusmc.nl>; Guoying Zhou <g.zhou@erasmusmc.nl>; Ruby Lieshout <r.lieshout@erasmusmc.nl>; Gilles van Tienderen <g.vantienderen@erasmusmc.nl>; valeska@glycostem.com; Martin van Royen <m.vanroyen@erasmusmc.nl>; Kübra Koten <k.koten@erasmusmc.nl>; y.y.kan@amsterdamumc.nl; 'Cecile Geuijen <C.Geuijen@merus.nl> (C.Geuijen@merus.nl)' (C.Geuijen@merus.nl) <C.Geuijen@merus.nl>; Dave Sprengers <d.sprengers@erasmusmc.nl>; Luc van der Laan <l.vanderlaan@erasmusmc.nl>; luciacampos3190@gmail.com; gezhouhong37@hotmail.com
Cc: Maikel Peppelenbosch <m.peppelenbosch@erasmusmc.nl>; magreluc <magreluc@gmail.com>; Patrick Boor <p.boor@erasmusmc.nl>
Subject: RE: Important! Please respond asap / acceptance manuscript BJC

Dear all,

I agree to the changes made in the manuscript BJC-A3339175R1.

Kind regards,
Jyaysi

From: Monique Verstegen <m.verstegen@erasmusmc.nl>
Sent: Tuesday, April 19, 2022 5:48 PM
To: Guoying Zhou <g.zhou@erasmusmc.nl>; Ruby Lieshout <r.lieshout@erasmusmc.nl>; Gilles van Tienderen <g.vantienderen@erasmusmc.nl>; valeska@glycostem.com; Martin van Royen <m.vanroyen@erasmusmc.nl>; Kübra Koten <k.koten@erasmusmc.nl>; y.y.kan@amsterdamumc.nl; 'Cecile Geuijen <C.Geuijen@merus.nl> (C.Geuijen@merus.nl)' (C.Geuijen@merus.nl) <C.Geuijen@merus.nl>; Dave Sprengers <d.sprengers@erasmusmc.nl>; Luc van der Laan <l.vanderlaan@erasmusmc.nl>; Monique Verstegen <m.verstegen@erasmusmc.nl>; luciacampos3190@gmail.com; gezhouhong37@hotmail.com
Cc: Maikel Peppelenbosch <m.peppelenbosch@erasmusmc.nl>; magreluc <magreluc@gmail.com>; Patrick Boor <p.boor@erasmusmc.nl>; Jyaysi Desai <j.desai@erasmusmc.nl>
Subject: Important! Please respond asap / acceptance manuscript BJC
Importance: High

Dear co-authors,

We are nearly there! Manuscript: BJC-A3339175R1 Modelling immune cytotoxicity for cholangiocarcinoma with tumor-derived organoids and effector T cells., gave us a small hick up that is fortunately easily addressed.

As we needed to do more experiments in absence of Ruby and Estella, we asked Luc (M), Patrick and Jyaysi if they could help us. And they did! So, all were included as co-authors on this manuscript. The Journal now would like to formally ask all of you if you agree to those changes. For this I would need an e-mail from you, in which you confirm that you agree.

Can you please send me your reply to this e-mail, including a statement that you agree to the changes made, i.e. including Luc Magre, Patrick P.C. Boor and Jyaysi Desai, in the author list at you soonest so I can bundle all replies and upload them to BJC.

The BJC also wanted me to contact you to ask you to link your own ORCID to this manuscript. You can do this via the author portal here: https://mts-bjcancer.nature.com/cgi-bin/main.plex and click on the 'Modify My Springer Nature Account' (see below)

General Tasks
Modify My Springer Nature Account *Click here to view your submitted manuscripts, article download*
Logout

Hope to see your reply soon,

With kind regards,
Monique Verstegen
Corresponding author Manuscript: BJC-A3339175R1

Dr. M.M.A (Monique) Verstegen Ph.D

Assistant professor

Surgery

+31 10 703 5528 |
m.verstegen@erasmusmc.nl
www.erasmusmc.nl

Dr. Molewaterplein 40, 3015 GD Rotterdam
P.O. Box 2040, 3000 CA Rotterdam

Monique Verstegen

From: Luc van der Laan
Sent: Tuesday, April 19, 2022 6:50 PM
To: Monique Verstegen
Cc: Maikel Peppelenbosch; magreluc; Patrick Boor; Jyaysi Desai
Subject: RE: Important! Please respond asap / acceptance manuscript BJC

Follow Up Flag: Flag for follow up
Flag Status: Flagged

Hi Monique,

I agree to the changes made, i.e. including Luc Magre, Patrick P.C. Boor and Jyaysi Desai, in the author list.

With kind regards
Luc van der Laan

Luc JW van der Laan, PhD
Professor in Liver Regenerative Medicine
Head of Laboratory LETIS

Erasmus MC - University Medical Center
Department of Surgery
Dr. Molewaterplein 40, Room Na-1008
3015 GD, Rotterdam
The Netherlands
Phone: +31 10 703 7557
Email assist: a.overgaauw@erasmusmc.nl
Website:Laboratorium LETIS

Dyslexia Warning! Apologies in advance for poor spelling.

The information in this e-mail and any Attachment is strictly confidential and is intended solely for the individual or company to whom it is addressed.

From: Monique Verstegen <m.verstegen@erasmusmc.nl>
Sent: dinsdag 19 april 2022 17:48
To: Guoying Zhou <g.zhou@erasmusmc.nl>; Ruby Lieshout <r.lieshout@erasmusmc.nl>; Gilles van Tienderen <g.vantienderen@erasmusmc.nl>; valeska@glycostem.com; Martin van Royen <m.vanroyen@erasmusmc.nl>; Kübra Koten <k.koten@erasmusmc.nl>; y.y.kan@amsterdamumc.nl; 'Cecile Geuijen <C.Geuijen@merus.nl> (C.Geuijen@merus.nl)' (C.Geuijen@merus.nl) <C.Geuijen@merus.nl>; Dave Sprengers <d.sprengers@erasmusmc.nl>; Luc van der Laan <l.vanderlaan@erasmusmc.nl>; Monique Verstegen <m.verstegen@erasmusmc.nl>; luciacampos3190@gmail.com; gezhouhong37@hotmail.com
Cc: Maikel Peppelenbosch <m.peppelenbosch@erasmusmc.nl>; magreluc <magreluc@gmail.com>; Patrick Boor <p.boor@erasmusmc.nl>; Jyaysi Desai <j.desai@erasmusmc.nl>
Subject: Important! Please respond asap / acceptance manuscript BJC
Importance: High

Dear co-authors,

We are nearly there! Manuscript: BJC-A3339175R1 Modelling immune cytotoxicity for cholangiocarcinoma with tumor-derived organoids and effector T cells., gave us a small hick up that is fortunately easily addressed.

As we needed to do more experiments in absence of Ruby and Estella, we asked Luc (M), Patrick and Jyaysi if they could help us. And they did! So, all were included as co-authors on this manuscript. The Journal now would like to formally ask all of you if you agree to those changes. For this I would need an e-mail from you, in which you confirm that you agree.

Can you please send me your reply to this e-mail, including a statement that you agree to the changes made, i.e. including Luc Magre, Patrick P.C. Boor and Jyaysi Desai, in the author list at you soonest so I can bundle all replies and upload them to BJC.

The BJC also wanted me to contact you to ask you to link your own ORCID to this manuscript. You can do this via the author portal here: https://mts-bjcancer.nature.com/cgi-bin/main.plex and click on the 'Modify My Springer Nature Account' (see below)

General Tasks
Modify My Springer Nature Account *Click here to view your submitted manuscripts, article download*
Logout

Hope to see your reply soon,

With kind regards,
Monique Verstegen
Corresponding author Manuscript: BJC-A3339175R1

Dr. M.M.A (Monique) Verstegen Ph.D

Assistant professor

Surgery

+31 10 703 5528 |
m.verstegen@erasmusmc.nl
www.erasmusmc.nl

Dr. Molewaterplein 40, 3015 GD Rotterdam
P.O. Box 2040, 3000 CA Rotterdam

Monique Verstegen

From: Lucia Campos <luciacampos3190@gmail.com>
Sent: Wednesday, April 20, 2022 9:08 AM
To: Monique Verstegen
Subject: Re: Important! Please respond asap / acceptance manuscript BJC

Follow Up Flag: Follow up
Flag Status: Completed

Waarschuwing: Deze e-mail is afkomstig van buiten de organisatie. Klik niet op links en open geen bijlagen, tenzij u de afzender herkent en weet dat de inhoud veilig is.
Caution: This email originated from outside of the organization. Do not click links or open attachments unless you recognize the sender and know the content is safe.

Dear Monique,

I agree with adding Luc Magre, Patrick P.C. Boor and Jyaysi Desai as co-authors to the manuscript BJC-A3339175R1.

Let me know if there is anything else needed from my side.

Best,
Lucia

--

Lucia Campos Carrascosa, Ph.D.

Brandstrasse 25
8952 Schlieren, CH
Tel. 0767251439

Am Di., 19. Apr. 2022 um 17:48 Uhr schrieb Monique Verstegen <m.verstegen@erasmusmc.nl>:

> Dear co-authors,
>
> We are nearly there! Manuscript: BJC-A3339175R1 Modelling immune cytotoxicity for cholangiocarcinoma with tumor-derived organoids and effector T cells., gave us a small hick up that is fortunately easily addressed.
>
> As we needed to do more experiments in absence of Ruby and Estella, we asked Luc (M), Patrick and Jyaysi if they could help us. And they did! So, all were included as co-authors on this manuscript. The Journal now would like to formally ask all of you if you agree to those changes. For this I would need an e-mail from you, in which you confirm that you agree.

Can you please send me your reply to this e-mail, including a statement that you agree to the changes made, i.e. including Luc Magre, Patrick P.C. Boor and Jyaysi Desai, in the author list at you soonest so I can bundle all replies and upload them to BJC.

The BJC also wanted me to contact you to ask you to link your own ORCID to this manuscript. You can do this via the author portal here: https://mts-bjcancer.nature.com/cgi-bin/main.plex and click on the 'Modify My Springer Nature Account' (see below)

General Tasks
Modify My Springer Nature Account *Click here to view your submitted manuscripts, article download statist*
Logout

Hope to see your reply soon,

With kind regards,

Monique Verstegen

Corresponding author Manuscript: BJC-A3339175R1

Dr. M.M.A (Monique) Verstegen Ph.D

Assistant professor

Surgery

+31 10 703 5528 |
m.verstegen@erasmusmc.nl
www.erasmusmc.nl

Dr. Molewaterplein 40, 3015 GD Rotterdam
P.O. Box 2040, 3000 CA Rotterdam

Monique Verstegen

From: Maikel Peppelenbosch
Sent: Thursday, April 21, 2022 9:00 AM
To: Monique Verstegen
Subject: RE: Important! Please respond asap / acceptance manuscript BJC

Dear Monique,

As the representative of Jaap Kwekkeboom I have the pleasure to inform you that you agree to the changes made, including Luc Magre, Patrick P.C. Boor and Jyaysi Desai, in the author list.

Met vriendelijke groet,

prof. dr. Maikel P. Peppelenbosch

Hoofd Laboratorium voor Maag-, Darm-, Leverziekten

Maag-, Darm-, Leverziekten

Van: Monique Verstegen <m.verstegen@erasmusmc.nl>
Verzonden: dinsdag 19 april 2022 17:48
Aan: Guoying Zhou <g.zhou@erasmusmc.nl>; Ruby Lieshout <r.lieshout@erasmusmc.nl>; Gilles van Tienderen <g.vantienderen@erasmusmc.nl>; valeska@glycostem.com; Martin van Royen <m.vanroyen@erasmusmc.nl>; Kübra Koten <k.koten@erasmusmc.nl>; y.y.kan@amsterdamumc.nl; 'Cecile Geuijen <C.Geuijen@merus.nl> (C.Geuijen@merus.nl)' (C.Geuijen@merus.nl) <C.Geuijen@merus.nl>; Dave Sprengers <d.sprengers@erasmusmc.nl>; Luc van der Laan <l.vanderlaan@erasmusmc.nl>; Monique Verstegen <m.verstegen@erasmusmc.nl>; luciacampos3190@gmail.com; gezhouhong37@hotmail.com
CC: Maikel Peppelenbosch <m.peppelenbosch@erasmusmc.nl>; magreluc <magreluc@gmail.com>; Patrick Boor <p.boor@erasmusmc.nl>; Jyaysi Desai <j.desai@erasmusmc.nl>
Onderwerp: Important! Please respond asap / acceptance manuscript BJC
Urgentie: Hoog

Dear co-authors,

We are nearly there! Manuscript: BJC-A3339175R1 Modelling immune cytotoxicity for cholangiocarcinoma with tumor-derived organoids and effector T cells., gave us a small hick up that is fortunately easily addressed.

As we needed to do more experiments in absence of Ruby and Estella, we asked Luc (M), Patrick and Jyaysi if they could help us. And they did! So, all were included as co-authors on this manuscript. The Journal now would like to formally ask all of you if you agree to those changes. For this I would need an e-mail from you, in which you confirm that you agree.

Can you please send me your reply to this e-mail, including a statement that you agree to the changes made, i.e. including Luc Magre, Patrick P.C. Boor and Jyaysi Desai, in the author list at you soonest so I can bundle all replies and upload them to BJC.

The BJC also wanted me to contact you to ask you to link your own ORCID to this manuscript. You can do this via the author portal here: https://mts-bjcancer.nature.com/cgi-bin/main.plex and click on the 'Modify My Springer Nature Account' (see below)

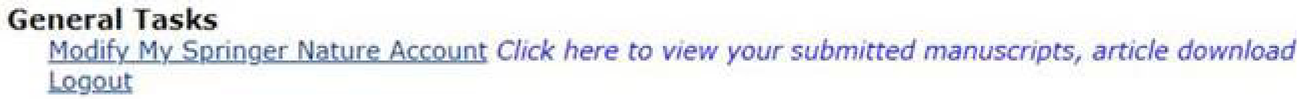

Hope to see your reply soon,

With kind regards,
Monique Verstegen
Corresponding author Manuscript: BJC-A3339175R1

Dr. M.M.A (Monique) Verstegen Ph.D

Assistant professor

Surgery

+31 10 703 5528 |
m.verstegen@erasmusmc.nl
www.erasmusmc.nl

Dr. Molewaterplein 40, 3015 GD Rotterdam
P.O. Box 2040, 3000 CA Rotterdam

Monique Verstegen

**From:**	Martin van Royen
**Sent:**	Wednesday, April 20, 2022 10:07 PM
**To:**	Monique Verstegen; Guoying Zhou; Ruby Lieshout; Gilles van Tienderen; valeska@glycostem.com; Kübra Koten; y.y.kan@amsterdamumc.nl; 'Cecile Geuijen <C.Geuijen@merus.nl> (C.Geuijen@merus.nl)' (C.Geuijen@merus.nl); Dave Sprengers; Luc van der Laan; luciacampos3190@gmail.com; gezhouhong37 @hotmail.com
**Cc:**	Maikel Peppelenbosch; magreluc; Patrick Boor; Jyaysi Desai
**Subject:**	Re: Important! Please respond asap / acceptance manuscript BJC
**Follow Up Flag:**	Flag for follow up
**Flag Status:**	Flagged

Dear Monique, I do agree with the changes made to the manuscript, including the addition of Luc Magre, Patrick P.C. Boor and Jyaysi Desai to the author list.

Best,
Martin E. van Royen

Martin E. van Royen, PhD
Assistant professor

Department of Pathology, Erasmus MC

Mail address; P.O. Box 2040, 3000 CA Rotterdam, The Netherlands
Visiting address; Office: H Be 0346, Wytemaweg 80, 3015 CN Rotterdam, The Netherlands
Tel: +31 10 7037644, Mobile: +31 6 12039870

E-mail: m.vanroyen@erasmusmc.nl

Van: Monique Verstegen <m.verstegen@erasmusmc.nl>
Verzonden: dinsdag 19 april 2022 17:48
Aan: Guoying Zhou <g.zhou@erasmusmc.nl>; Ruby Lieshout <r.lieshout@erasmusmc.nl>; Gilles van Tienderen <g.vantienderen@erasmusmc.nl>; valeska@glycostem.com <valeska@glycostem.com>; Martin van Royen <m.vanroyen@erasmusmc.nl>; Kübra Koten <k.koten@erasmusmc.nl>; y.y.kan@amsterdamumc.nl <y.y.kan@amsterdamumc.nl>; 'Cecile Geuijen <C.Geuijen@merus.nl> (C.Geuijen@merus.nl)' (C.Geuijen@merus.nl) <C.Geuijen@merus.nl>; Dave Sprengers <d.sprengers@erasmusmc.nl>; Luc van der Laan <l.vanderlaan@erasmusmc.nl>; Monique Verstegen <m.verstegen@erasmusmc.nl>; luciacampos3190@gmail.com <luciacampos3190@gmail.com>; gezhouhong37@hotmail.com <gezhouhong37@hotmail.com>
CC: Maikel Peppelenbosch <m.peppelenbosch@erasmusmc.nl>; magreluc <magreluc@gmail.com>; Patrick Boor <p.boor@erasmusmc.nl>; Jyaysi Desai <j.desai@erasmusmc.nl>
Onderwerp: Important! Please respond asap / acceptance manuscript BJC

Dear co-authors,

We are nearly there! Manuscript: BJC-A3339175R1 Modelling immune cytotoxicity for cholangiocarcinoma with tumor-derived organoids and effector T cells., gave us a small hick up that is fortunately easily addressed.

As we needed to do more experiments in absence of Ruby and Estella, we asked Luc (M), Patrick and Jyaysi if they could help us. And they did! So, all were included as co-authors on this manuscript. The Journal now would like to

formally ask all of you if you agree to those changes. For this I would need an e-mail from you, in which you confirm that you agree.

Can you please send me your reply to this e-mail, including a statement that you agree to the changes made, i.e. including Luc Magre, Patrick P.C. Boor and Jyaysi Desai, in the author list at you soonest so I can bundle all replies and upload them to BJC.

The BJC also wanted me to contact you to ask you to link your own ORCID to this manuscript. You can do this via the author portal here: https://mts-bjcancer.nature.com/cgi-bin/main.plex and click on the 'Modify My Springer Nature Account' (see below)

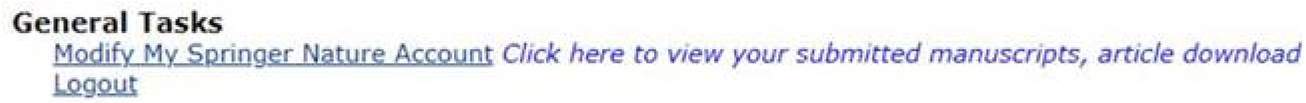

Hope to see your reply soon,

With kind regards,
Monique Verstegen
Corresponding author Manuscript: BJC-A3339175R1

Dr. M.M.A (Monique) Verstegen Ph.D

Assistant professor

Surgery

+31 10 703 5528 |
m.verstegen@erasmusmc.nl
www.erasmusmc.nl

Dr. Molewaterplein 40, 3015 GD Rotterdam
P.O. Box 2040, 3000 CA Rotterdam

Monique Verstegen

From: Patrick Boor
Sent: Wednesday, April 20, 2022 10:14 AM
To: Monique Verstegen
Subject: RE: Important! Please respond asap / acceptance manuscript BJC

Hi Monique,

I confirm that I agree to the changes that are made to the paper

Regards,

Patrick Boor

Van: Monique Verstegen <m.verstegen@erasmusmc.nl>
Verzonden: dinsdag 19 april 2022 17:48
Aan: Guoying Zhou <g.zhou@erasmusmc.nl>; Ruby Lieshout <r.lieshout@erasmusmc.nl>; Gilles van Tienderen <g.vantienderen@erasmusmc.nl>; valeska@glycostem.com; Martin van Royen <m.vanroyen@erasmusmc.nl>; Kübra Koten <k.koten@erasmusmc.nl>; y.y.kan@amsterdamumc.nl; 'Cecile Geuijen <C.Geuijen@merus.nl> (C.Geuijen@merus.nl)' (C.Geuijen@merus.nl) <C.Geuijen@merus.nl>; Dave Sprengers <d.sprengers@erasmusmc.nl>; Luc van der Laan <l.vanderlaan@erasmusmc.nl>; Monique Verstegen <m.verstegen@erasmusmc.nl>; luciacampos3190@gmail.com; gezhouhong37@hotmail.com
CC: Maikel Peppelenbosch <m.peppelenbosch@erasmusmc.nl>; magreluc <magreluc@gmail.com>; Patrick Boor <p.boor@erasmusmc.nl>; Jyaysi Desai <j.desai@erasmusmc.nl>
Onderwerp: Important! Please respond asap / acceptance manuscript BJC
Urgentie: Hoog

Dear co-authors,

We are nearly there! Manuscript: BJC-A3339175R1 Modelling immune cytotoxicity for cholangiocarcinoma with tumor-derived organoids and effector T cells., gave us a small hick up that is fortunately easily addressed.

As we needed to do more experiments in absence of Ruby and Estella, we asked Luc (M), Patrick and Jyaysi if they could help us. And they did! So, all were included as co-authors on this manuscript. The Journal now would like to formally ask all of you if you agree to those changes. For this I would need an e-mail from you, in which you confirm that you agree.

Can you please send me your reply to this e-mail, including a statement that you agree to the changes made, i.e. including Luc Magre, Patrick P.C. Boor and Jyaysi Desai, in the author list at you soonest so I can bundle all replies and upload them to BJC.

The BJC also wanted me to contact you to ask you to link your own ORCID to this manuscript. You can do this via the author portal here: https://mts-bjcancer.nature.com/cgi-bin/main.plex and click on the 'Modify My Springer Nature Account' (see below)

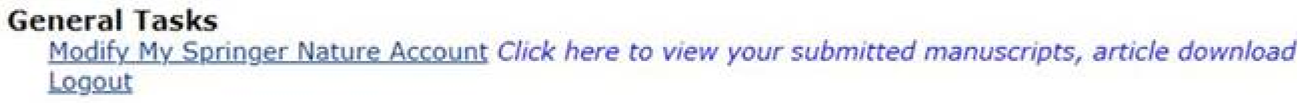

Hope to see your reply soon,

With kind regards,
Monique Verstegen
Corresponding author Manuscript: BJC-A3339175R1

Dr. M.M.A (Monique) Verstegen Ph.D

Assistant professor

Surgery

+31 10 703 5528 |
m.verstegen@erasmusmc.nl
www.erasmusmc.nl

Dr. Molewaterplein 40, 3015 GD Rotterdam
P.O. Box 2040, 3000 CA Rotterdam

Erasmus MC TRANSPLANT INSTITUTE

Monique Verstegen

From: Ruby Lieshout
Sent: Tuesday, April 19, 2022 6:00 PM
To: Monique Verstegen
Subject: RE: Important! Please respond asap / acceptance manuscript BJC

Follow Up Flag: Follow up
Flag Status: Flagged

Dear Monique,

I agree to the addition of the new co-authors Luc Magre, Patrick P.C. Boor and Jyaysi Desai to the author list of this manuscript (BJC-A3339175R1).

Kind regards,

R Lieshout

PhD Student

Surgery
Erasmus MC Transplant Institute

r.lieshout@erasmusmc.nl
www.erasmusmc.nl

Dr. Molewaterplein 40, 3015 GD Rotterdam
P.O. Box 2040, 3000 CA Rotterdam

Erasmus MC

From: Monique Verstegen <m.verstegen@erasmusmc.nl>
Sent: dinsdag 19 april 2022 17:48
To: Guoying Zhou <g.zhou@erasmusmc.nl>; Ruby Lieshout <r.lieshout@erasmusmc.nl>; Gilles van Tienderen <g.vantienderen@erasmusmc.nl>; valeska@glycostem.com; Martin van Royen <m.vanroyen@erasmusmc.nl>; Kübra Koten <k.koten@erasmusmc.nl>; y.y.kan@amsterdamumc.nl; 'Cecile Geuijen <C.Geuijen@merus.nl> (C.Geuijen@merus.nl)' (C.Geuijen@merus.nl) <C.Geuijen@merus.nl>; Dave Sprengers <d.sprengers@erasmusmc.nl>; Luc van der Laan <l.vanderlaan@erasmusmc.nl>; Monique Verstegen <m.verstegen@erasmusmc.nl>; luciacampos3190@gmail.com; gezhouhong37@hotmail.com
Cc: Maikel Peppelenbosch <m.peppelenbosch@erasmusmc.nl>; magreluc <magreluc@gmail.com>; Patrick Boor <p.boor@erasmusmc.nl>; Jyaysi Desai <j.desai@erasmusmc.nl>
Subject: Important! Please respond asap / acceptance manuscript BJC
Importance: High

Dear co-authors,

We are nearly there! Manuscript: BJC-A3339175R1 Modelling immune cytotoxicity for cholangiocarcinoma with tumor-derived organoids and effector T cells., gave us a small hick up that is fortunately easily addressed.

As we needed to do more experiments in absence of Ruby and Estella, we asked Luc (M), Patrick and Jyaysi if they could help us. And they did! So, all were included as co-authors on this manuscript. The Journal now would like to

formally ask all of you if you agree to those changes. For this I would need an e-mail from you, in which you confirm that you agree.

<u>Can you please send me your reply to this e-mail, including a statement that you agree to the changes made, i.e. including Luc Magre, Patrick P.C. Boor and Jyaysi Desai, in the author list at you soonest</u> so I can bundle all replies and upload them to BJC.

The BJC also wanted me to contact you to ask you to link your own ORCID to this manuscript. You can do this via the author portal here: https://mts-bjcancer.nature.com/cgi-bin/main.plex and click on the 'Modify My Springer Nature Account' (see below)

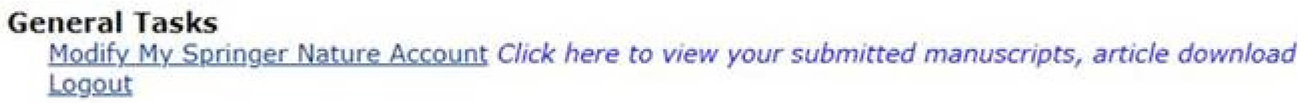

Hope to see your reply soon,

With kind regards,
Monique Verstegen
Corresponding author Manuscript: BJC-A3339175R1

Dr. M.M.A (Monique) Verstegen Ph.D

Assistant professor

Surgery

+31 10 703 5528 |
m.verstegen@erasmusmc.nl
www.erasmusmc.nl

Dr. Molewaterplein 40, 3015 GD Rotterdam
P.O. Box 2040, 3000 CA Rotterdam

Erasmus MC Transplant Institute

Monique Verstegen

From: Valeska de Ruiter <valeska@glycostem.com>
Sent: Thursday, April 21, 2022 4:32 PM
To: Monique Verstegen
Subject: RE: Important! Please respond asap / acceptance manuscript BJC

Follow Up Flag: Flag for follow up
Flag Status: Flagged

Waarschuwing: Deze e-mail is afkomstig van buiten de organisatie. Klik niet op links en open geen bijlagen, tenzij u de afzender herkent en weet dat de inhoud veilig is.
Caution: This email originated from outside of the organization. Do not click links or open attachments unless you recognize the sender and know the content is safe.

Dear Monique,

I agree to the changes made in the manuscript BJC-A3339175R1, i.e. including Luc Magre, Patrick P.C. Boor and Jyaysi Desai, in the author list.

Kind regards,
Valeska de Ruiter

Valeska de Ruiter
Development Technician

glycostem

T: +31 880 025 909
F: +31 412 712 900
Kloosterstraat 9
5349 AB Oss, The Netherlands
www.Glycostem.com

My working days are Monday to Friday

This email and any files transmitted with it are confidential and intended solely for the use of the individual or entity to whom they are addressed. If you are not the intended recipient you are notified that disclosing, copying, distributing or taking any action in reliance on the contents of this information is strictly prohibited.

From: Monique Verstegen <m.verstegen@erasmusmc.nl>
Sent: Tuesday, April 19, 2022 5:48 PM
To: Guoying Zhou <g.zhou@erasmusmc.nl>; Ruby Lieshout <r.lieshout@erasmusmc.nl>; Gilles van Tienderen <g.vantienderen@erasmusmc.nl>; Valeska de Ruiter <valeska@glycostem.com>; Martin van Royen

<m.vanroyen@erasmusmc.nl>; Kübra Koten <k.koten@erasmusmc.nl>; y.y.kan@amsterdamumc.nl; 'Cecile Geuijen <C.Geuijen@merus.nl> (C.Geuijen@merus.nl)' (C.Geuijen@merus.nl) <C.Geuijen@merus.nl>; Dave Sprengers <d.sprengers@erasmusmc.nl>; Luc van der Laan <l.vanderlaan@erasmusmc.nl>; Monique Verstegen <m.verstegen@erasmusmc.nl>; luciacampos3190@gmail.com; gezhouhong37@hotmail.com
Cc: Maikel Peppelenbosch <m.peppelenbosch@erasmusmc.nl>; magreluc <magreluc@gmail.com>; Patrick Boor <p.boor@erasmusmc.nl>; Jyaysi Desai <j.desai@erasmusmc.nl>
Subject: Important! Please respond asap / acceptance manuscript BJC
Importance: High

> You don't often get email from m.verstegen@erasmusmc.nl. Learn why this is important

Dear co-authors,

We are nearly there! Manuscript: BJC-A3339175R1 Modelling immune cytotoxicity for cholangiocarcinoma with tumor-derived organoids and effector T cells., gave us a small hick up that is fortunately easily addressed.

As we needed to do more experiments in absence of Ruby and Estella, we asked Luc (M), Patrick and Jyaysi if they could help us. And they did! So, all were included as co-authors on this manuscript. The Journal now would like to formally ask all of you if you agree to those changes. For this I would need an e-mail from you, in which you confirm that you agree.

Can you please send me your reply to this e-mail, including a statement that you agree to the changes made, i.e. including Luc Magre, Patrick P.C. Boor and Jyaysi Desai, in the author list at you soonest so I can bundle all replies and upload them to BJC.

The BJC also wanted me to contact you to ask you to link your own ORCID to this manuscript. You can do this via the author portal here: https://mts-bjcancer.nature.com/cgi-bin/main.plex and click on the 'Modify My Springer Nature Account' (see below)

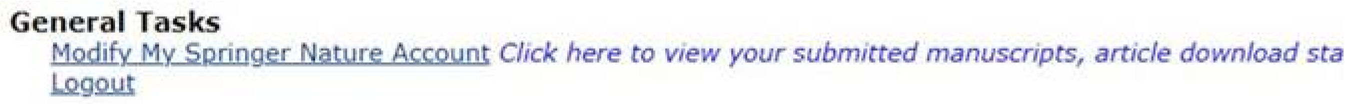

Hope to see your reply soon,

With kind regards,
Monique Verstegen
Corresponding author Manuscript: BJC-A3339175R1

Dr. M.M.A (Monique) Verstegen Ph.D

Assistant professor

Surgery

+31 10 703 5528 |
m.verstegen@erasmusmc.nl
www.erasmusmc.nl

Dr. Molewaterplein 40, 3015 GD Rotterdam
P.O. Box 2040, 3000 CA Rotterdam

Monique Verstegen

From: Kan, Y.Y. (Yik) <y.y.kan@amsterdamumc.nl>
Sent: Tuesday, April 19, 2022 9:59 PM
To: Monique Verstegen
Subject: RE: Important! Please respond asap / acceptance manuscript BJC

Follow Up Flag: Flag for follow up
Flag Status: Flagged

Waarschuwing: Deze e-mail is afkomstig van buiten de organisatie. Klik niet op links en open geen bijlagen, tenzij u de afzender herkent en weet dat de inhoud veilig is.
Caution: This email originated from outside of the organization. Do not click links or open attachments unless you recognize the sender and know the content is safe.

Hi Monique,

Hierbij bevestig ik, dat ik hiermee akkoord ga.

Met vriendelijke groetjes,
Yik

Van: Monique Verstegen <m.verstegen@erasmusmc.nl>
Verzonden: dinsdag 19 april 2022 17:48
Aan: Guoying Zhou <g.zhou@erasmusmc.nl>; Ruby Lieshout <r.lieshout@erasmusmc.nl>; Gilles van Tienderen <g.vantienderen@erasmusmc.nl>; valeska@glycostem.com; Martin van Royen <m.vanroyen@erasmusmc.nl>; Kübra Koten <k.koten@erasmusmc.nl>; Kan, Y.Y. (Yik) <y.y.kan@amsterdamumc.nl>; 'Cecile Geuijen <C.Geuijen@merus.nl> (C.Geuijen@merus.nl)' (C.Geuijen@merus.nl) <C.Geuijen@merus.nl>; Dave Sprengers <d.sprengers@erasmusmc.nl>; Luc van der Laan <l.vanderlaan@erasmusmc.nl>; Monique Verstegen <m.verstegen@erasmusmc.nl>; luciacampos3190@gmail.com; gezhouhong37@hotmail.com
CC: Maikel Peppelenbosch <m.peppelenbosch@erasmusmc.nl>; magreluc <magreluc@gmail.com>; Patrick Boor <p.boor@erasmusmc.nl>; Jyaysi Desai <j.desai@erasmusmc.nl>
Onderwerp: Important! Please respond asap / acceptance manuscript BJC
Urgentie: Hoog

Dear co-authors,

We are nearly there! Manuscript: BJC-A3339175R1 Modelling immune cytotoxicity for cholangiocarcinoma with tumor-derived organoids and effector T cells., gave us a small hick up that is fortunately easily addressed.

As we needed to do more experiments in absence of Ruby and Estella, we asked Luc (M), Patrick and Jyaysi if they could help us. And they did! So, all were included as co-authors on this manuscript. The Journal now would like to formally ask all of you if you agree to those changes. For this I would need an e-mail from you, in which you confirm that you agree.

Can you please send me your reply to this e-mail, including a statement that you agree to the changes made, i.e. including Luc Magre, Patrick P.C. Boor and Jyaysi Desai, in the author list at you soonest so I can bundle all replies and upload them to BJC.

The BJC also wanted me to contact you to ask you to link your own ORCID to this manuscript. You can do this via the author portal here: https://mts-bjcancer.nature.com/cgi-bin/main.plex and click on the 'Modify My Springer Nature Account' (see below)

General Tasks

Modify My Springer Nature Account *Click here to view your submitted manuscripts, article download*

Logout

Hope to see your reply soon,

With kind regards,
Monique Verstegen
Corresponding author Manuscript: BJC-A3339175R1

Dr. M.M.A (Monique) Verstegen Ph.D

Assistant professor

Surgery

+31 10 703 5528 |
m.verstegen@erasmusmc.nl
www.erasmusmc.nl

Dr. Molewaterplein 40, 3015 GD Rotterdam
P.O. Box 2040, 3000 CA Rotterdam

VUmc disclaimer : www.vumc.nl/disclaimer
AMC disclaimer : www.amc.nl/disclaimer

Monique Verstegen

From: Zhouhong Ge <gezhouhong37@hotmail.com>
Sent: Wednesday, April 20, 2022 12:18 PM
To: Monique Verstegen
Subject: RE: Important! Please respond asap / acceptance manuscript BJC

Waarschuwing: Deze e-mail is afkomstig van buiten de organisatie. Klik niet op links en open geen bijlagen, tenzij u de afzender herkent en weet dat de inhoud veilig is.
Caution: This email originated from outside of the organization. Do not click links or open attachments unless you recognize the sender and know the content is safe.

Dear Monique,

I agree to the changes made in the manuscript BJC-A3339175R1.

Best regards,
Zhouhong

From: Kübra Koten
Sent: Wednesday, April 20, 2022 10:45 AM
To: Jyaysi Desai; Monique Verstegen; Guoying Zhou; Ruby Lieshout; Gilles van Tienderen; valeska@glycostem.com; Martin van Royen; y.y.kan@amsterdamumc.nl; 'Cecile Geuijen <C.Geuijen@merus.nl> (C.Geuijen@merus.nl)' (C.Geuijen@merus.nl); Dave Sprengers; Luc van der Laan; luciacampos3190@gmail.com; gezhouhong37@hotmail.com
Cc: Maikel Peppelenbosch; magreluc; Patrick Boor
Subject: RE: Important! Please respond asap / acceptance manuscript BJC

Dear all,

I agree to the changes made in the manuscript BJC-A3339175R1.

Kind regards,

Kübra Köten

From: Jyaysi Desai <j.desai@erasmusmc.nl>
Sent: Wednesday, April 20, 2022 10:22
To: Monique Verstegen <m.verstegen@erasmusmc.nl>; Guoying Zhou <g.zhou@erasmusmc.nl>; Ruby Lieshout <r.lieshout@erasmusmc.nl>; Gilles van Tienderen <g.vantienderen@erasmusmc.nl>; valeska@glycostem.com; Martin van Royen <m.vanroyen@erasmusmc.nl>; Kübra Koten <k.koten@erasmusmc.nl>; y.y.kan@amsterdamumc.nl; 'Cecile Geuijen <C.Geuijen@merus.nl> (C.Geuijen@merus.nl)' (C.Geuijen@merus.nl) <C.Geuijen@merus.nl>; Dave Sprengers <d.sprengers@erasmusmc.nl>; Luc van der Laan <l.vanderlaan@erasmusmc.nl>; luciacampos3190@gmail.com; gezhouhong37@hotmail.com
Cc: Maikel Peppelenbosch <m.peppelenbosch@erasmusmc.nl>; magreluc <magreluc@gmail.com>; Patrick Boor <p.boor@erasmusmc.nl>
Subject: RE: Important! Please respond asap / acceptance manuscript BJC

Dear all,

I agree to the changes made in the manuscript BJC-A3339175R1.

Kind regards,
Jyaysi

From: Monique Verstegen <m.verstegen@erasmusmc.nl>
Sent: Tuesday, April 19, 2022 5:48 PM
To: Guoying Zhou <g.zhou@erasmusmc.nl>; Ruby Lieshout <r.lieshout@erasmusmc.nl>; Gilles van Tienderen <g.vantienderen@erasmusmc.nl>; valeska@glycostem.com; Martin van Royen <m.vanroyen@erasmusmc.nl>; Kübra Koten <k.koten@erasmusmc.nl>; y.y.kan@amsterdamumc.nl; 'Cecile Geuijen <C.Geuijen@merus.nl> (C.Geuijen@merus.nl)' (C.Geuijen@merus.nl) <C.Geuijen@merus.nl>; Dave Sprengers <d.sprengers@erasmusmc.nl>; Luc van der Laan <l.vanderlaan@erasmusmc.nl>; Monique Verstegen <m.verstegen@erasmusmc.nl>; luciacampos3190@gmail.com; gezhouhong37@hotmail.com
Cc: Maikel Peppelenbosch <m.peppelenbosch@erasmusmc.nl>; magreluc <magreluc@gmail.com>; Patrick Boor <p.boor@erasmusmc.nl>; Jyaysi Desai <j.desai@erasmusmc.nl>
Subject: Important! Please respond asap / acceptance manuscript BJC
Importance: High

Dear co-authors,

We are nearly there! Manuscript: BJC-A3339175R1 Modelling immune cytotoxicity for cholangiocarcinoma with tumor-derived organoids and effector T cells., gave us a small hick up that is fortunately easily addressed.

As we needed to do more experiments in absence of Ruby and Estella, we asked Luc (M), Patrick and Jyaysi if they could help us. And they did! So, all were included as co-authors on this manuscript. The Journal now would like to formally ask all of you if you agree to those changes. For this I would need an e-mail from you, in which you confirm that you agree.

Can you please send me your reply to this e-mail, including a statement that you agree to the changes made, i.e. including Luc Magre, Patrick P.C. Boor and Jyaysi Desai, in the author list at you soonest so I can bundle all replies and upload them to BJC.

The BJC also wanted me to contact you to ask you to link your own ORCID to this manuscript. You can do this via the author portal here: https://mts-bjcancer.nature.com/cgi-bin/main.plex and click on the 'Modify My Springer Nature Account' (see below)

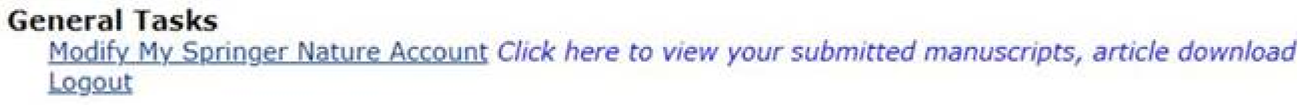

Hope to see your reply soon,

With kind regards,
Monique Verstegen
Corresponding author Manuscript: BJC-A3339175R1

Dr. M.M.A (Monique) Verstegen Ph.D

Assistant professor

Surgery

+31 10 703 5528 |
m.verstegen@erasmusmc.nl
www.erasmusmc.nl

Dr. Molewaterplein 40, 3015 GD Rotterdam
P.O. Box 2040, 3000 CA Rotterdam

Erasmus MC
TRANSPLANT INSTITUTE
